# Triglyceride-Glucose Index Is Not Associated With Lung Cancer Risk: A Prospective Cohort Study in the UK Biobank

**DOI:** 10.3389/fonc.2021.774937

**Published:** 2021-11-17

**Authors:** Lijie Wang, Shucheng Si, Jiqing Li, Yunxia Li, Xiaolu Chen, Fuzhong Xue, Wangang Ren

**Affiliations:** ^1^ Department of Epidemiology and Health Statistics, School of Public Health, Cheeloo College of Medicine, Shandong University, Jinan, China; ^2^ Institute for Medical Dataology, Shandong University, Jinan, China; ^3^ Department of Thoracic Surgery, Shandong Provincial Hospital, Cheeloo College of Medicine, Shandong University, Jinan, China

**Keywords:** lung cancer, insulin resistance, UK Biobank, longitudinal study, triglyceride-glucose index

## Abstract

**Background:**

The triglyceride-glucose (TyG) index is a practical substitute measure for insulin resistance (IR). The relationship between IR and lung cancer has been examined in previous studies; however, the findings have been controversial. In addition, previous studies had small sample sizes. Thus, we systematically examined the association between IR and lung cancer risk based on the UK Biobank with IR measured by the TyG index and further examined the interactions and joint effects for lung cancer.

**Methods:**

A total of 324,334 individuals free from any type of cancer at recruitment from the UK Biobank prospective cohort were included. The participants were predominantly between 40 and 70 years old. After adjusting for relevant confounders, multivariable Cox regression models were constructed to examine the relationship between the TyG index and the risk of lung cancer. We also checked the interactions and joint effects using a polygenic risk score (PRS) for lung cancer.

**Results:**

During a median follow-up of 9 years, 1,593 individuals were diagnosed with lung cancer. No association was found between the TyG index and lung cancer risk after multivariate Cox regression analysis adjusted for risk factors (hazard ratio: 0.91; 95% confidence interval: 0.64–1.18). No interaction or joint effects for genetic risk and the TyG index were observed.

**Conclusion:**

The TyG index was not associated with the risk of lung cancer. Our results provide limited evidence that IR is not correlated with the risk of lung cancer.

## Introduction

Lung cancer is one of the most commonly diagnosed cancers causing many deaths each year (representing approximately one in 10 cancers diagnosed and one in five deaths in 2020) (1). Despite improved treatment, the diagnosis of lung cancer is associated with relatively poor survival (2). Identifying the population at high risk of lung cancer remains an arduous task (3).

Insulin resistance (IR) is one of the most common metabolic disorders (4, 5). The triglyceride-glucose index (TyG index), a surrogate indicator of combined triglycerides (TGs) and glucose, is considered to be a practical and effective measurement for IR (6–8). Some studies have shown that the TyG index plays an important role as a potential risk factor for some diseases, such as metabolic syndrome (9), acute pancreatitis (10), cardio-cerebrovascular diseases (11), and cancers of the digestive system (12). Although some previous studies have preliminarily examined the relationship between IR and lung cancer, the evidence is unconvincing and somewhat controversial. Several studies have shown that IR is positively related to lung cancer, whereas some studies found an invalid association (13–17). In addition, most of the literature had a small sample size and insufficient estimates of genetic predisposition. Whether IR can assist in predicting and diagnosing lung cancer remains unclear. Although genetic susceptibility alleles could explain approximately 12% of heritability for lung cancer (18), and more than 50 genetic susceptibility loci have been identified in different ethnic groups (19), no work has been done to investigate the joint effects or interactions between the TyG index and the genetic susceptibility for lung cancer.

In this study, we sought to systematically examine the association between IR and lung cancer risk based on the UK Biobank, with IR measured by the TyG index, and further examined the interactions and joint effects using a polygenic risk score (PRS) for lung cancer. 

## Materials and Methods

### Study Cohort

We used data from the UK Biobank, which is an ongoing population-based national prospective cohort study. Approximately half a million people between 37 and 73 years of age were recruited for the study across the United Kingdom from 2006 to 2010 (20, 21). At baseline assessment, participants completed a standardized questionnaire that included detailed information on socioeconomic and demographic characteristics, general health and medical information, lifestyle, and diet. Physical assessments, laboratory investigations, and genome-wide genotyping of all participants were also performed at baseline. Written informed consent was obtained from all participants, and the study protocol was approved by the North West Multi-Center Research Ethics Committee, the Patient Information Advisory Group in England and Wales, and the Community Health Index Advisory Group in Scotland.

### Ascertainment of Exposures

Demographic data on age, sex, region, socioeconomic status (Townsend deprivation score), smoking status and alcohol intake frequency were obtained by administering a standardized questionnaire. Region refers to the UK Biobank Assessment Center at which the participant consented (22). Material deprivation was measured using the Townsend Index. Each participant was assigned a score corresponding to the output area in which their postcode was located. Smoking status was categorized as never, former, or current smoking. Alcohol intake frequency was categorized as never, special occasions only, 1-3 times/month, 1-2 times/week, 3 or 4 times/week, or daily or almost daily. Body mass index (BMI) was calculated by dividing body weight (kg) by height in meters squared (m^2^). For analyses, BMI was categorized into normal (<25 kg/m^2^), overweight (25–29.9 kg/m^2^), and obese (≥30 kg/m^2^). The waist-hip ratio (WHR) was calculated by dividing waist circumference by hip circumference.

Blood samples were collected at recruitment of participants into UK Biobank, and taken at random, and the time (hours) since the last meal and the fasting time were recorded during collection (23). Data on TG, glucose, total cholesterol (TC), high-density lipoprotein cholesterol (HDL), low-density lipoprotein cholesterol (LDL), and glycated hemoglobin (HbA1c), including fasting time before sampling, were collected during health examinations. The TyG index was calculated using the formula: *ln* [*triglyceride* (*mg*/*dL*) × *glucose* (*mg*/*dL*)/2]. (24) The HbA1c value was categorized into normal and high with a clinical cutoff of ≥42 mmol/mol (25, 26). Diabetes (International Classification of Diseases, 10th Revision [ICD-10]: E10-E14), dyslipidemia (ICD-10: E78), and hypertension (ICD-10: I10-I15) were defined from the health records of the UK Biobank. Further details on the definition of the selected variables are provided in **Table S1**.

### Selection Criteria

**Figure 1:**
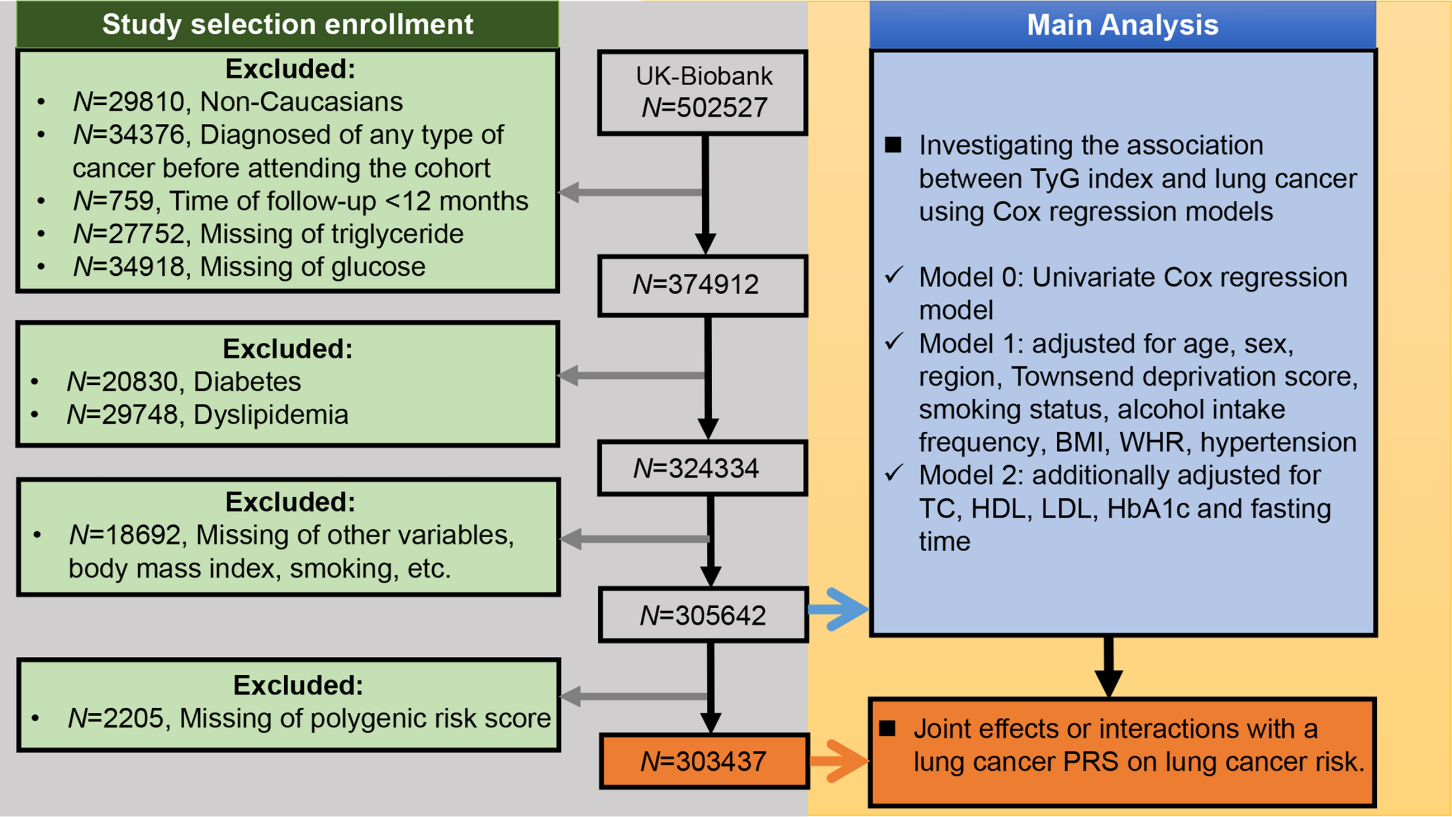
Selection criteria for building the lung cancer cohort and the main analysis of this study. BMI, body mass index; WHR, waist-hip ratio; TC, total cholesterol; TyG index, triglyceride-glucose index; HDL, high-density lipoprotein cholesterol; LDL, low-density lipoprotein cholesterol; HbA1c, glycated hemoglobin; PRS, polygenic risk score.

We included participants who met the following criteria: 1) Caucasian; 2) no previous history of any type of cancer before enrollment; 3) without diabetes or dyslipidemia; and 4) no missing data. Further, we excluded participants with a follow-up time of less than 1 year from the study to minimize the possibility of reverse causality (i.e., parameters of interest affected by undiagnosed cancer) (24). **Figure 1** presents a flowchart of the study selection process.

### Polygenic Risk Score Construction

Details of the genotyping and imputation procedures for single nucleotide polymorphisms (SNPs) in the UK Biobank can be found elsewhere (27). Eighteen SNPs that were significantly associated with lung cancer risk (*P* < 5×10^-8^) in Caucasians in the study by McKay et al. were identified from a genome-wide association study (**Table S2**) (18). The PRS for lung cancer was constructed for each participant by summing the risk allele numbers (i.e., participants have 0, 1, or 2 risk alleles) weighted by their respective effect sizes [*β* coefficients, as natural log-transformed values (odds ratios)] (28). The effect of missing SNP observations was set to a value of zero.

### Ascertainment of Outcome

The study outcome was incident diagnosis of lung cancer of any of the topographic subcategories, recorded *via* linkages to national cancer and death registries according to the ICD-10 code C34. Participants were followed up from the date of recruitment until the date of lung cancer diagnosis, the date of death, or 15 February 2018, whichever came first. 

### Statistical Analysis

Means ± standard deviations (SD) or medians (interquartile ranges) were calculated for continuous variables, whereas frequencies and proportions (*N*, %) were reported for categorical variables. Quantitative baseline variables were compared using the t-test. Categorical variables were compared between groups using the chi-square test.

Cox regression models were used to estimate hazard ratios (HRs) and 95% confidence intervals (CIs) for the risk of lung cancer according to the TyG index increment. A univariate Cox regression model (Model 0) was constructed in the first step. Subsequently, a multivariate Cox regression model (Model 1) was adjusted for age at recruitment (continuous), sex, region (10 categories: London, North-West, North-East, East Midlands, West Midlands, South-East, South-West, Wales, Yorkshire and Humber, Scotland), Townsend deprivation score (quartile), smoking status (never, former, current), alcohol intake frequency (6 categories: never, special occasions only, 1-3 times/month, 1-2 times/week, 3 or 4 times/week, and daily or almost daily), BMI (normal, overweight, obese), WHR (quartile), and hypertension (yes, no). Furthermore, Model 2 was additionally adjusted for TC (continuous), HDL (continuous), LDL (continuous), HbA1c (normal, high), and fasting time (continuous). Covariates were selected based on scientific plausibility and previous studies. Restricted cubic spline models were fitted to Cox proportional hazards models (29). Information about the data collection of covariates is provided in **Table S1**. The main analysis of this study is presented in **Figure 1**.

Furthermore, the continuous variable TyG index was divided into categories, and the above three Cox models were applied as described previously. First, the TyG index was divided by deciles, and the three Cox models were used to determine the overall trend of HRs for the TyG index with lung cancer risk. In the subsequent analysis, we categorized participants as having low-level and high-level TyG indices according to the median. Stratum-specific analyses were conducted to assess the potential effects of age group at recruitment (<55, 55-64, or ≥65 years), sex, Townsend deprivation score (tertiles), smoking status (never, former, or current), alcohol intake frequency (never, less than daily, daily or almost daily), BMI (normal, overweight, obese), WHR (tertiles), TC (tertiles), HDL (tertiles), LDL (tertiles), HbA1c (tertiles), fasting time (<8, ≥ 8 hours), and hypertension (yes, no).

Analyses of the genetic susceptibility of lung cancer were restricted to participants with a complete lung cancer PRS (**Figure 1**). We assessed the statistical significance of the potential effect modifications by interaction testing using likelihood ratio tests. Participants were divided into categories of low (quintile 1), intermediate (quintiles 2–4), and high (quintile 5) PRS (28). The effects of the TyG index were detected at each genetic level, and the joint effects were assessed in different genetic risk groups.

We conducted a sensitivity analysis by removing participants whose TyG index was beyond the range of mean ± 3SD. We also did the sensitivity analysis to examine the robustness of our results: removing participants with a follow-up time of less than 2 years. All analyses were performed using R version 4.0.3 (http://www.rproject.org/), and a two-tailed *P-*value < 0.05 was considered statistically significant.

## Results

Among the 502,527 UK Biobank participants, 29,810 were non-Caucasians, 34,376 had a pre-baseline diagnosis of any cancer, and 759 had less than 12 months of follow-up. Meanwhile, 27,752 and 34,918 participants were excluded because they were missing TG and glucose values, respectively. We also excluded 20,830 participants with diabetes and 29,748 participants with dyslipidemia (**Figure 1**). Among the remaining 324,334 UK Biobank participants, there were 1,593 incident lung cancer diagnoses during a median follow-up of 9.07 years (interquartile range: 8.34–9.74 years). The median age was 57 (range: 38-73) years old.

### TyG Index and Baseline Characteristics

Baseline demographic and clinical characteristics are listed in **Table 1**. There were significant differences between the two groups in terms of age at recruitment, region, sex, smoking status, alcohol intake frequency, and hypertension (all *P* < 0.01). The TyG index was higher in the lung cancer group than in the group without lung cancer (*P* < 0.01). A significant excess risk of lung cancer was observed for Townsend deprivation score, WHR, fasting time, HbA1c and TG levels (all *P* < 0.01). However, TC, HDL-C, and LDL-C levels were significantly lower in patients with lung cancer than in those without lung cancer (all *P* < 0.01). The BMI and glucose levels were similar between the two groups (*P* = 0.817 and 0.296, respectively).

**Table 1:** Baseline demographic and clinical characteristics in the study.

Characteristics	Level	No Lung Cancer (n = 322741)	Lung Cancer (n = 1593)	Total (n = 324334)	*P* value
Age	mean (sd) median	55.805 (8.051) 57	61.08 (6.121) 62	55.831 (8.051) 57	<0.001
Sex	Female	180,245 (55.848)	812 (50.973)	181,057 (55.824)	<0.001
Sex	Male	142496 (44.152)	781 (49.027)	143,277 (44.176)	
Region	London	38,499 (11.929)	144 (9.040)	38,643 (11.915)	<0.001
Region	Wales	14300 (4.431)	64 (4.018)	14,364 (4.429)	
Region	North West	46327 (14.354)	279 (17.514)	46,606 (14.370)	
Region	North East	37438 (11.600)	196 (12.304)	37,634 (11.603)	
Region	Yorkshier and Humber	49346 (15.290)	218 (13.685)	49,564 (15.282)	
Region	West Midlands	28198 (8.737)	118 (7.407)	28,316 (8.731)	
Region	East Midlands	22512 (6.975)	98 (6.152)	22,610 (6.971)	
Region	South East	29875 (9.257)	131 (8.223)	30,006 (9.252)	
Region	South West	29594 (9.170)	135 (8.475)	29,729 (9.166)	
Region	Scotland	26652 (8.258)	210 (13.183)	26,862 (8.282)	
Townsend deprivation index	[-6.26,-3.73]	80,731 (25.044)	281 (17.640)	81,012 (25.008)	<0.001
Townsend deprivation index	(-3.73,-2.32]	80663 (25.023)	300 (18.832)	80,963 (24.993)	
Townsend deprivation index	(-2.32,0.128]	80614 (25.008)	375 (23.540)	80,989 (25.001)	
Townsend deprivation index	(0.128,11]	80347 (24.925)	637 (39.987)	80,984 (24.999)	
Townsend deprivation index	missing	386	0	386	
BMI	Normal	114,815 (35.673)	575 (36.369)	115,390 (35.676)	0.817
BMI	Overweight	137889 (42.842)	674 (42.631)	138,563 (42.841)	
BMI	Obese	69154 (21.486)	332 (20.999)	69,486 (21.483)	
BMI	missing	883	12	895	
WHR	<=0.796	80,958 (25.126)	251 (15.816)	81,209 (25.080)	<0.001
WHR	(0.796,0.864]	80432 (24.963)	365 (22.999)	80,797 (24.953)	
WHR	(0.864,0.927]	80633 (25.025)	430 (27.095)	81,063 (25.035)	
WHR	>0.927	80185 (24.886)	541 (34.089)	80,726 (24.931)	
WHR	missing	533	6	539	
Smoking status	Never	180,665 (56.154)	269 (16.993)	180,934 (55.962)	<0.001
Smoking status	Previous	108576 (33.747)	649 (40.998)	109,225 (33.783)	
Smoking status	Current	32492 (10.099)	665 (42.009)	33,157 (10.255)	
Smoking status	missing	1008	10	1018	
Alcohol intake frequency	Never	19,558 (6.064)	156 (9.805)	19,714 (6.082)	<0.001
Alcohol intake frequency	Special occasions only	32815 (10.174)	191 (12.005)	33,006 (10.183)	
Alcohol intake frequency	1-3 Times/month	36153 (11.209)	136 (8.548)	36,289 (11.196)	
Alcohol intake frequency	1-2 Times/week	86049 (26.680)	372 (23.382)	86,421 (26.664)	
Alcohol intake frequency	3 or 4 Times/week	79493 (24.647)	312 (19.610)	79,805 (24.623)	
Alcohol intake frequency	Daily or almost daily	68455 (21.225)	424 (26.650)	68,879 (21.251)	
Alcohol intake frequency	missing	218	2	220	
Hypertension	No	277,358 (85.938)	1,196 (75.078)	278,554 (85.885)	<0.001
Hypertension	Yes	45383 (14.062)	397 (24.922)	45,780 (14.115)	
Fasting time	mean (sd) median	3.754 (2.395) 3	4.11 (2.918) 3	3.755 (2.398) 3	<0.001
Fasting time	missing	8	0	8	
TG	mean (sd) median	1.692 (0.982) 1.437	1.812 (1.028) 1.574	1.692 (0.982) 1.438	<0.001
Glucose	mean (sd) median	4.987 (0.823) 4.9	4.965 (0.77) 4.901	4.987 (0.823) 4.9	0.296
TyG index	mean (sd) median	8.667 (0.541) 8.638	8.739 (0.529) 8.722	8.668 (0.541) 8.639	<0.001
TC	mean (sd) median	5.786 (1.087) 5.737	5.675 (1.124) 5.65	5.786 (1.087) 5.737	<0.001
TC	missing	79	0	79	
HDL	mean (sd) median	1.477 (0.38) 1.429	1.423 (0.386) 1.368	1.476 (0.38) 1.428	<0.001
HDL	missing	68	0	68	
LDL	mean (sd) median	3.624 (0.832) 3.582	3.55 (0.869) 3.534	3.623 (0.832) 3.582	<0.001
LDL	missing	455	1	456	
HbA1c	Normal	296,039 (96.406)	1,406 (92.744)	297,445 (96.388)	<0.001
HbA1c	High	11037 (3.594)	110 (7.256)	11,147 (3.612)	
HbA1c	missing	15665	77	15742	

sd, standard deviation; BMI, Body mass index; WHR, waist-hip ratio; TC, total cholesterol; TG, triglyceride; TyG index, Triglyceride-glucose index; HDL, high-density lipoprotein cholesterol; LDL, low-density lipoprotein cholesterol; HbA1c, glycated hemoglobin; TyG index, triglyceride-glucose index.

### TyG Index and Risk of Lung Cancer

When applying the univariate Cox model to the TyG index, a significant excess risk of lung cancer was observed (HR: 1.757, 95% CI: 1.518–1.995, *P* < 0.01). There was no evidence of an elevated risk of lung cancer linked to the TyG index in Model 1 (HR: 0.895, 95% CI: 0.644–1.145, *P* = 0.385) after adjusting for age, sex, region, Townsend deprivation score, smoking status, alcohol intake frequency, BMI, WHR, and hypertension. Similar estimates were obtained when fasting time, TC, LDL-C, HDL-C, and HbA1c were included in Model 2 (HR: 0.911, 95% CI: 0.640–1.182, *P* = 0.499) (**Table 2**). Furthermore, we did not observe significant associations between the TyG index grouped by deciles and lung cancer risk since almost all CIs crossed the line with the HR equal to 1 in both Models 1 and 2, except for the last group of TyG index (**Supplementary Figure 1**). When dividing the TyG index by median level (TyG index = 8.639), the results were the same as before with no significant associations in Model 1 (HR: 0.974, 95% CI: 0.876-1.083, *P* = 0.629) and Model 2 (HR: 0.966, 95% CI: 0.850–1.097, *P* = 0.589) (**Table 2**).

**Table 2:** Hazard ratios and 95% confidence intervals for lung cancer according to the TyG index.

Model	Beta	SE	HR (95%CI)	Wald	P
Model 0^a^	0.564	0.122	1.757 (1.518-1.995)	4.63	<0.001
Model 0^b^	0.259	0.051	1.295 (1.173-1.430)	5.123	<0.001
Model 1^a^	-0.111	0.128	0.895 (0.644–1.145)	-0.87	0.385
Model 1^b^	-0.026	0.054	0.974 (0.876-1.083)	-0.484	0.629
Model 2^a^	-0.093	0.138	0.911 (0.640-1.182)	-0.68	0.499
Model 2^b^	-0.035	0.065	0.966 (0.850-1.097)	-0.540	0.589

^a^and ^b^ represent the continuous type and categorial type (divided by median) of the TyG index respectively when we performed the Cox models. Model 0: univariate Cox models. Model 1: adjusted for age, sex, region, Townsend deprivation score, smoking status, alcohol intake frequency, body mass index, waist hip rate, and hypertension. Model 2: adjusted for model 1 plus fasting time, total cholesterol, low-density lipoprotein cholesterol, high-density lipoprotein cholesterol, and glycated hemoglobin.

### Subgroup Analysis and Sensitivity Analyses

In addition, we restricted our analyses to participants whose fasting time was ≥ 8 h, and the results were similar to the results for the entire study population (**Supplementary Figure 2**). Meanwhile, the subgroup analysis also showed consistent results in that no evidence of effect modification was observed for the TyG index with lung cancer risk in different groups since all CIs contained 1 in both Model 1 and Model 2 (**Supplementary Figure 2**). Sensitivity analyses showed that the multivariable-adjusted associations remained unchanged after excluding individuals whose TyG index was beyond the range of mean ± 3SD (**Supplementary Figure 3**) or whose follow-up time less than 2 years (**Supplementary Figure 4**).

### Joint Effects and Interactions for Lung Cancer According to PRS

**Figure 2:**
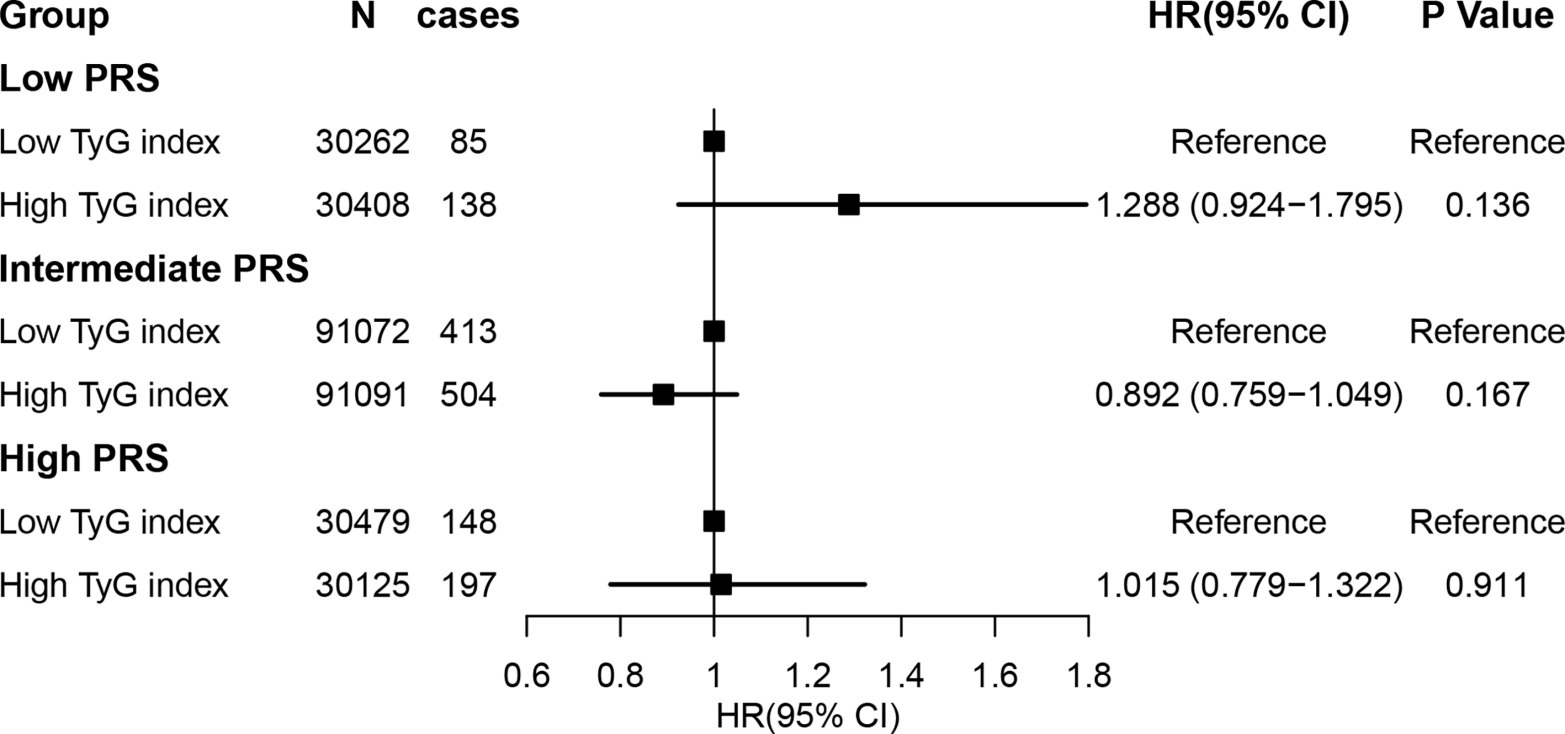
Risk of incident lung cancer according to genetic risk and the TyG index. Participants were divided into categories of low (quintile 1), intermediate (quintiles 2–4), and high (quintile 5) genetic risk strata. The TyG index was defined as low and high according to median level (TyG index = 8.639). The hazard ratios were estimated using Cox regression models after adjusting for age, sex, region, Townsend deprivation score, smoking status, alcohol intake frequency, body mass index, waist hip ratio, and hypertension, fasting time, total cholesterol, low-density lipoprotein cholesterol, high-density lipoprotein cholesterol, and glycated hemoglobin as well as the top 10 principal components of the ancestry and genotyping array. TyG index, triglyceride-glucose index.

We observed an increased risk of lung cancer in participants with higher lung cancer PRS (*N* = 320820, cases = 1579; adjusted HR per SD increase: 1.481; 95% CI: 1.300–1.687; **Supplementary Figure 5**), which is consistent with previous studies. The association of the TyG index with lung cancer was not significant after adjusting for PRS (HR: 1.193, 95% CI: 0.901–1.580, *P* = 0.218), and we did not identify a significant interaction between the TyG index and lung cancer (genetic score group [intermediate]: TyG index group [high], HR interaction: 0.767, 95% CI: 0.568–1.035, *P* = 0.083; and genetic score group [high]: TyG index group [high], HR interaction: 0.835, 95% CI: 0.592-1.178, *P* = 0.304) (also see **Figure 2** and **Supplementary Figure 6**). 

## Discussion

Based on a large-scale prospective cohort study, we systematically examined the association between IR quantified using the TyG index and incident lung cancer risk. Although a significant excess risk of lung cancer was observed for the TyG index in the lung cancer group or in the univariate Cox model, no association was found between the TyG index and lung cancer risk after multivariate Cox regression analysis adjusted for risk factors. Subgroup and sensitivity analyses confirmed the results. Since both genetic and environmental factors could collectively contribute to lung cancer risk, we studied the joint effects of the TyG index and PRS on lung cancer risk. The studied PRS comprised 18 significant SNPs obtained from a published genome-wide association study (18). Our study found no statistically significant interaction between the TyG index and the genetic propensity for lung cancer.

IR refers to a condition of impaired insulin action in promoting glucose uptake and use. A decline in insulin sensitivity can lead to a series of disorders (4, 5, 30). In the 1960s, it was observed that diabetes, obesity, lipid metabolism disorders, and hypertension often occur simultaneously in the same individual. In 1995, Stern proposed the “common soil” theory, which states that IR is the common basis for the above-mentioned diseases (31). IR can be assessed using various methods. The gold standard test for measuring insulin insensitivity is the euglycemic-hyperinsulinemic clamp in which peripheral glucose uptake is measured under conditions of elevated insulin concentrations. However, it is difficult to apply the glucose clamp in larger population studies and clinical settings because it is expensive and time-consuming (32). In recent years, studies have shown that the TyG index, calculated using TGs and glucose, has high sensitivity and specificity for identifying IR. Moreover, it is fast, inexpensive, and easy to use (6, 7).

It has been recognized that IR is closely related to cardio-cerebrovascular diseases. Our previous studies have confirmed that the TyG index is a sensitive pre-diagnostic indicator for cardio-cerebrovascular diseases (11). Other studies have also shown that the TyG index is closely related to cancers of the digestive organs and kidneys and that an increased BMI has a substantial effect on the risk of these cancers (24). However, very few studies have examined the association between the TyG index and lung cancer. A recent study reported that the TyG index is remarkably higher in patients with non-small cell lung cancer than in controls in a Chinese population (16). Consequently, the TyG index may be a suitable tumor marker for non-small cell lung cancer. The main purpose of our study was to explore whether the TyG index is a suitable predictor of lung cancer.

Previous studies investigating the association between lung cancer and IR have yielded inconsistent results. Some studies have shown that IR increases the risk of lung cancer (15, 33). Many mechanisms have been proposed to explain this observation. For example, elevated insulin levels may potentiate the activity of insulin-like growth factor-I, which represents a potent growth-promoting factor for lung cancer (34), and insulin may stimulate the Ras signaling pathway to promote lung carcinogenesis (35, 36). Moreover, additional mechanisms, such as stimulation of local angiogenesis or direct growth promotion *via* insulin receptors available on lung cancer cells, cannot be excluded. However, some studies, especially observational studies, have shown that IR is not associated with lung cancer, which is consistent with our results. The exact mechanism underlying this phenomenon is not yet clear. One possible view suggests that it is related to abnormal lipid metabolism in patients with tumors. Many cancers can cause fatty acid oxidation and/or adipose tissue lipolysis, and excessive fatty acids in the circulation may lead to IR. Abnormal lipid metabolism has been proposed as a cause of IR in obesity and type 2 diabetes (17, 30, 31). Han et al. reported an association between the blockage of whole-body fatty acid oxidation or adipose tissue lipolysis, whole-body IR, and glucose intolerance (37). IR may not be a tumor inducer but an extrapulmonary symptom in the development of lung cancer. Furthermore, several factors including obesity can independently modify both cancer risk and insulin resistance (38, 39). 1) Obesity is a worldwide health problem that is closely associated with IR and hyperinsulinemia (40). Many studies have found a strong correlation between obesity and IR (5, 41–43). 2) Obesity is a major risk factor for several common cancers (24, 44). However, a high BMI has been correlated with a reduced risk of lung cancer (45–47). 3) Hence, obesity may represent a confounding factor in the analyses of studies linking IR and lung cancer. In this study, consistent conclusions were reached after multivariate analysis adjusting the BMI and other relevant factors (**Table 2**), and in the subsequent stratification analysis by different levels of BMI (**Supplementary Figure 2**). Smoking is recognized as a major risk factor for lung cancer (3, 48). Smoking can suppress β-cell function, thereby reducing insulin secretion. A negative correlation between smoking and insulin has been observed in many epidemiological studies. This inverse relationship may also inhibit the tumor-promoting effects of hyperinsulinemia in lung cancer (49).

The exact mechanism between IR and lung cancer have not yet been fully understood, and more in-depth preclinical and clinical studies are needed to have a more detailed molecules and mechanisms understanding.

Our study is advantageous because of its design. First, this study had a large sample size and was prospectively designed. The longitudinal design could help reduce chance findings. The results from the multivariate Cox models at the continuous and categorical levels of the TyG index replicated each other to some degree and were further verified by incorporating the PRS of lung cancer. Second, to provide more stable and reliable results, subgroup analysis and sensitivity analyses were performed; however, the results remained the same. This somewhat reconfirmed that the analysis was stable and reliable.

Nevertheless, our study has some limitations. First, some information, such as physical activities, and female reproductive factors, was not included in the assessment, which could have affected the results. Second, we did not consider the confounding effect of inflammation, which could have overestimated the estimated indirect effect based on insulin. It has been demonstrated that the inflammatory milieu is associated with IR, obesity, dyslipidemia, and tumorigenesis (50). Third, the participants were all Caucasians from the UK Biobank, which may have contributed to selection bias while also affecting the generalizability of the results.

In conclusion, the present study showed that the TyG index level was not associated with the incidence of lung cancer. These results indicate that IR cannot identify the population at high risk of lung cancer, although the underlying mechanisms require further clarification.

## Data Availability Statement

Publicly available datasets were analyzed in this study. This data can be found here: This research has been conducted using the UK Biobank Resource (Application ID: 51470). Researchers may have access to the data by submitting an application to the UK Biobank (https://www.ukbiobank.ac.uk/) through the UK Biobank Access Management System (https://bbams.ndph.ox.ac.uk/ams/).

## Ethics Statement

The study protocol was approved by the North West Multi-center Research Ethics Committee, the Patient Information Advisory Group in England and Wales, and the Community Health Index Advisory Group in Scotland. All participants in the surveys have given informed consent. The patients/participants provided their written informed consent to participate in this study.

## Author Contributions

LW and WR drafted this manuscript. LW, SS, JL, YL, and XC performed the data analysis. FX and WR conceived of the study and participated in its design and coordination. WR contributed to the interpretation. All authors discussed the results and commented on the manuscript. All authors contributed to the article and approved the submitted version.

## Funding

This study was supported by the National Key Research and Development Program of China (2020YFC2003500), the National Natural Science Foundation of China (81773547), the Natural Science Foundation of Shandong Province (ZR2019ZD02), and Shandong Province Major Science and Technology Innovation Project (2018CXGC1210).

## Conflict of Interest

The authors declare that the research was conducted in the absence of any commercial or financial relationships that could be construed as a potential conflict of interest.

## Publisher’s Note

All claims expressed in this article are solely those of the authors and do not necessarily represent those of their affiliated organizations, or those of the publisher, the editors and the reviewers. Any product that may be evaluated in this article, or claim that may be made by its manufacturer, is not guaranteed or endorsed by the publisher.

## Abbreviations

BMI, body mass index; CI, confidence interval; HbA1c, glycated hemoglobin; HDL, high-density lipoprotein; HR, hazard ratio; ICD-10, International Classification of Diseases, 10th revision; IR, insulin resistance; LDL, low-density lipoprotein; PRS, polygenic risk score; SD, standard deviation; SNP, single nucleotide polymorphism; TC, total cholesterol; TG, triglyceride; TyG, triglyceride-glucose index; WHR, waist-hip ratio.
