# Supplementary material for: Triglyceride-Glucose Index Is Not Associated With Lung Cancer Risk: A Prospective Cohort Study in the UK Biobank
Source: Front Oncol. 2021 Nov 17;11:774937. doi: 10.3389/fonc.2021.774937 (PMC8635521; doi:10.3389/fonc.2021.774937)
Supplement: Supplementary file 1 [file DataSheet_1.docx]

Supplementary Material

# Supplementary Figures and Tables

## Supplementary Figures

**
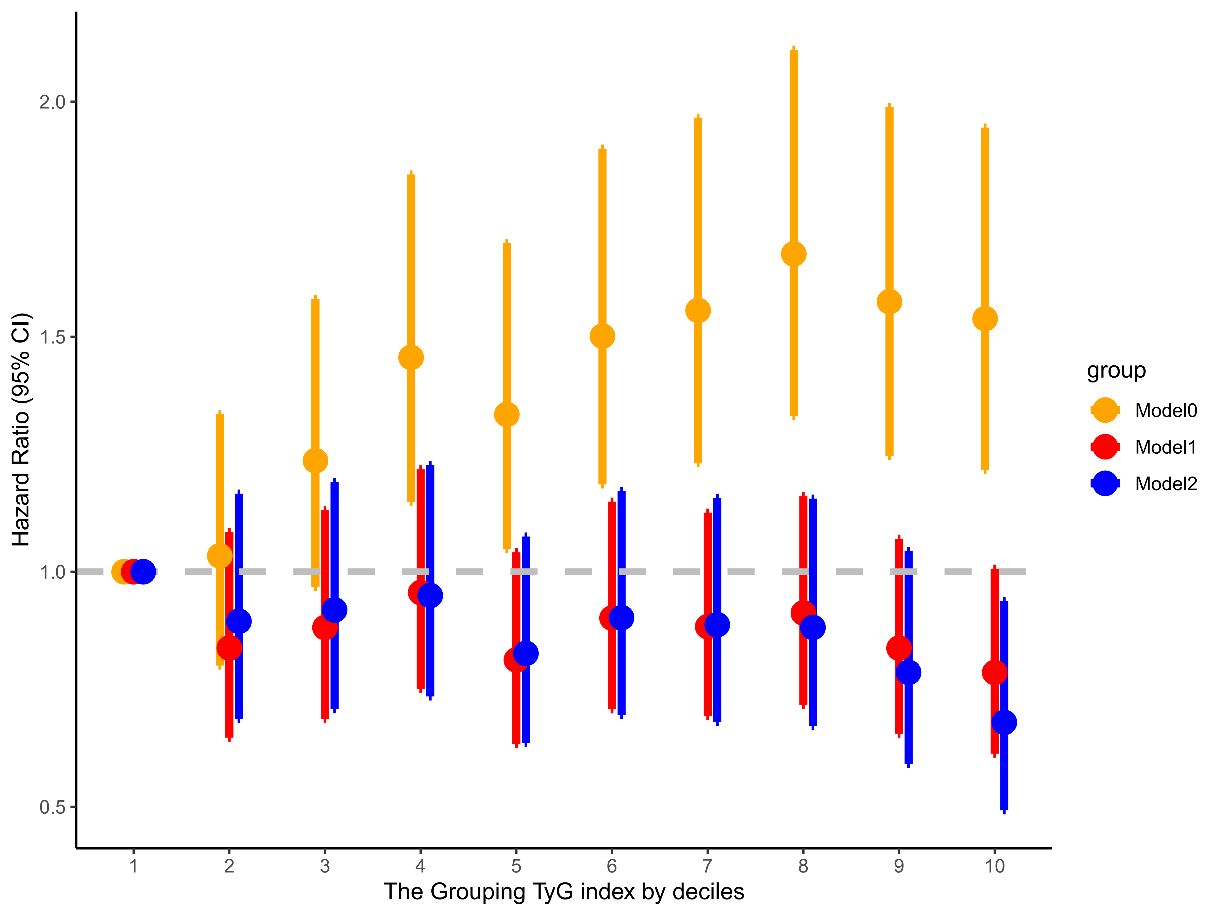
**

**Supplementary Figure 1.**

Hazard ratios and 95% confidence intervals of the triglyceride-glucose index divided by deciles with lung cancer risk. Model 0: univariate Cox model; Model 1: adjusted for age, sex, region, Townsend deprivation score, smoking status, alcohol intake frequency, body mass index, waist hip ratio, and hypertension; Model 2: adjusted for Model 1 plus fasting time, total cholesterol, low-density lipoprotein cholesterol, high-density lipoprotein cholesterol, and glycated hemoglobin.


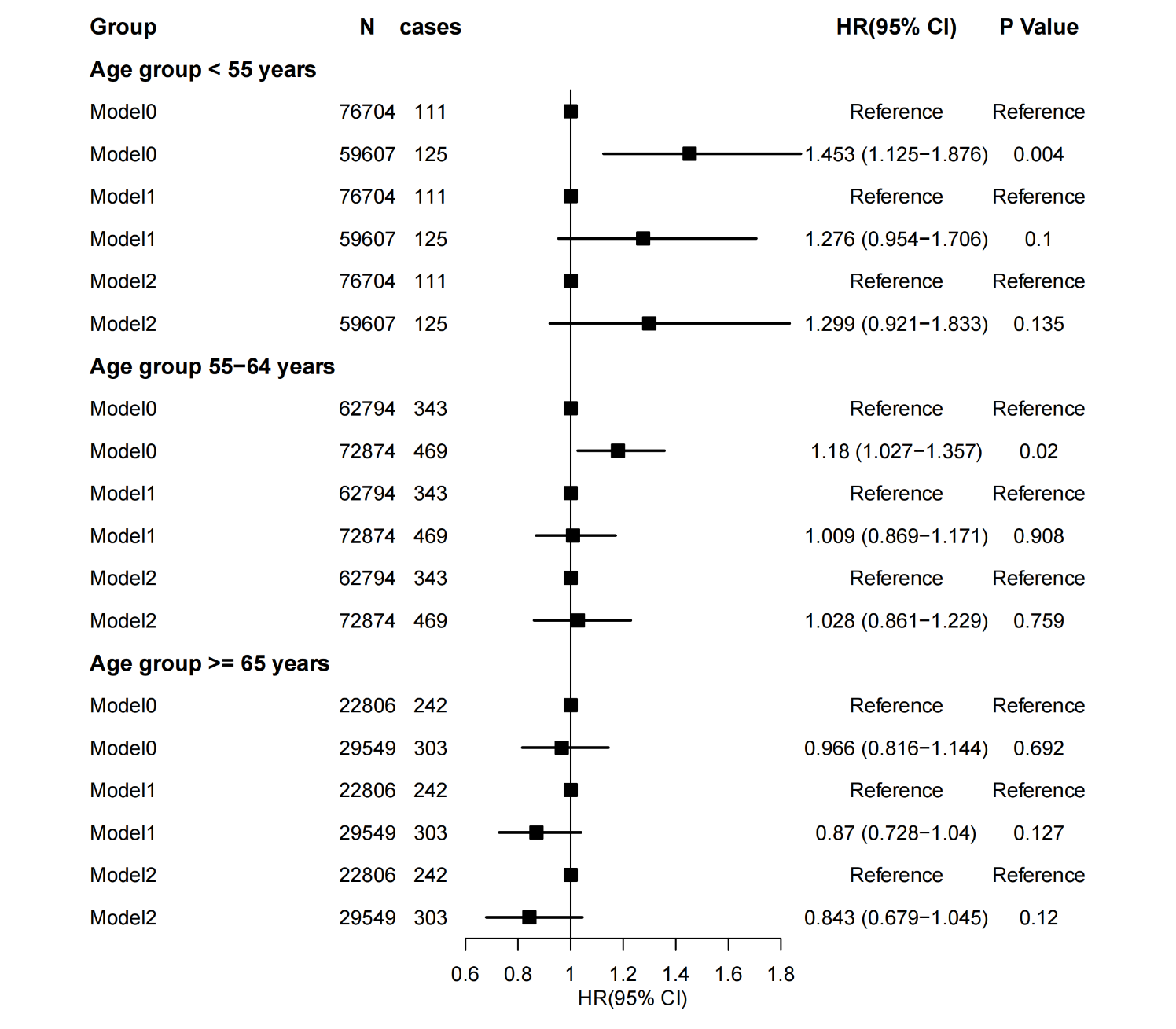

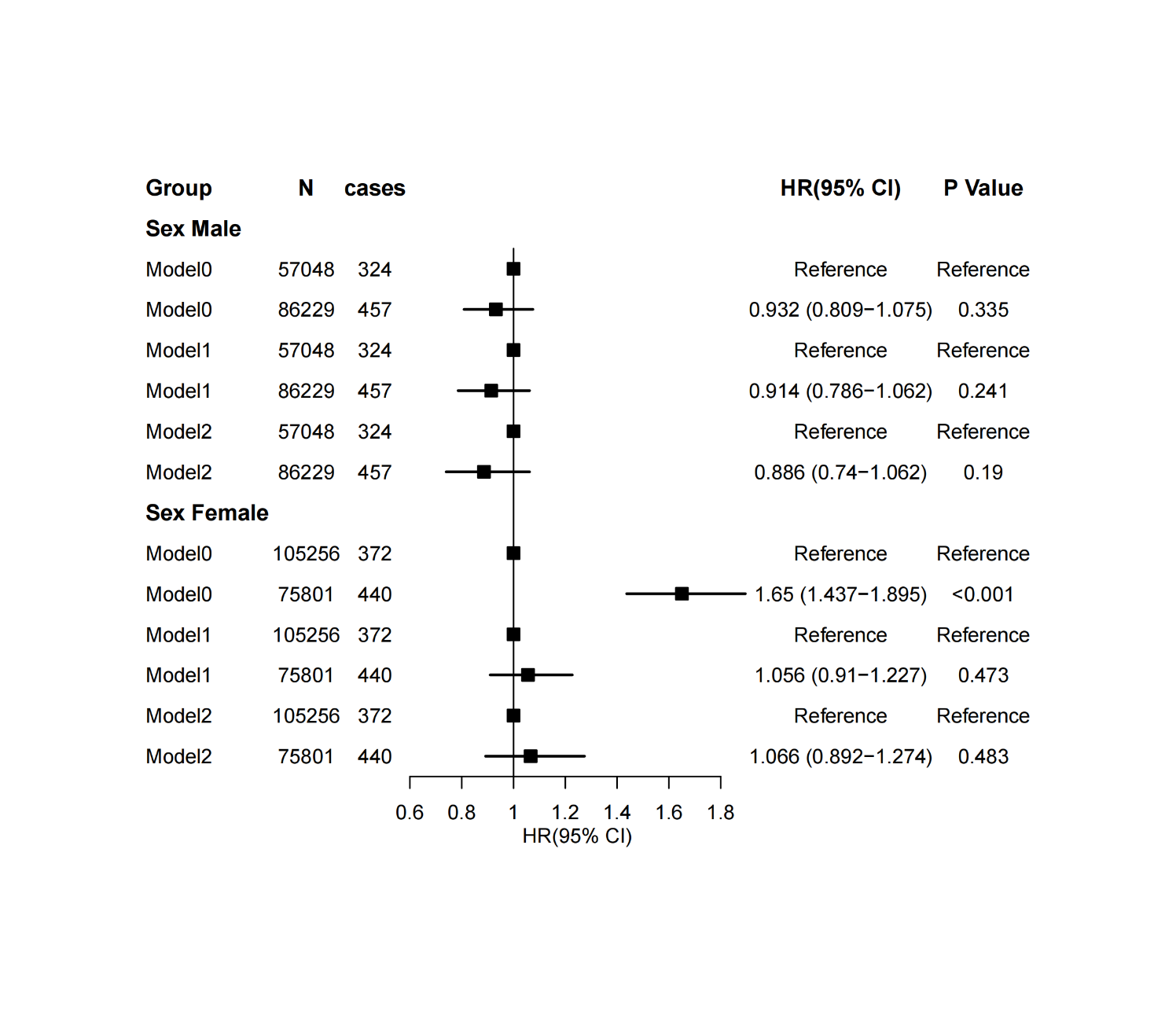

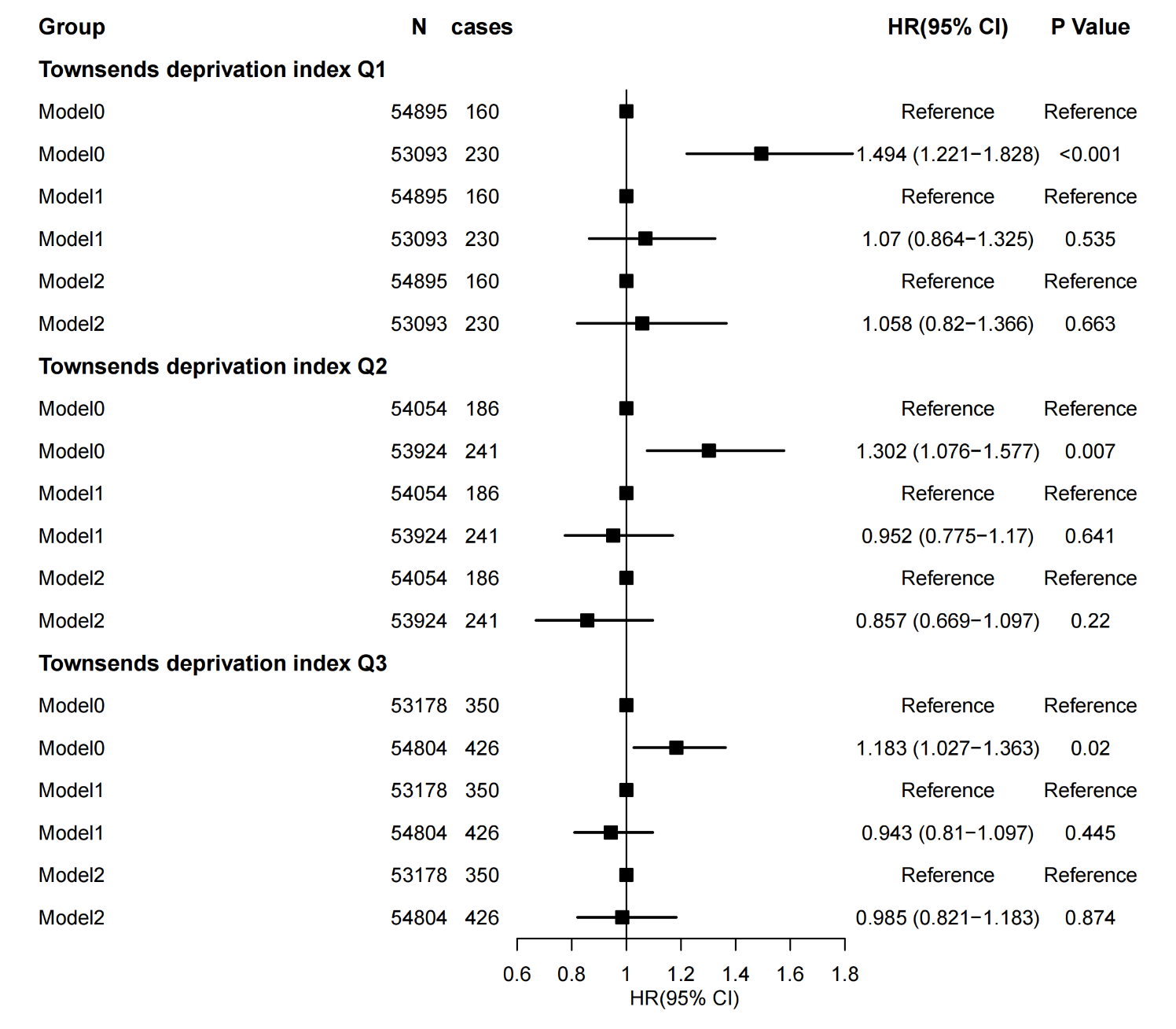

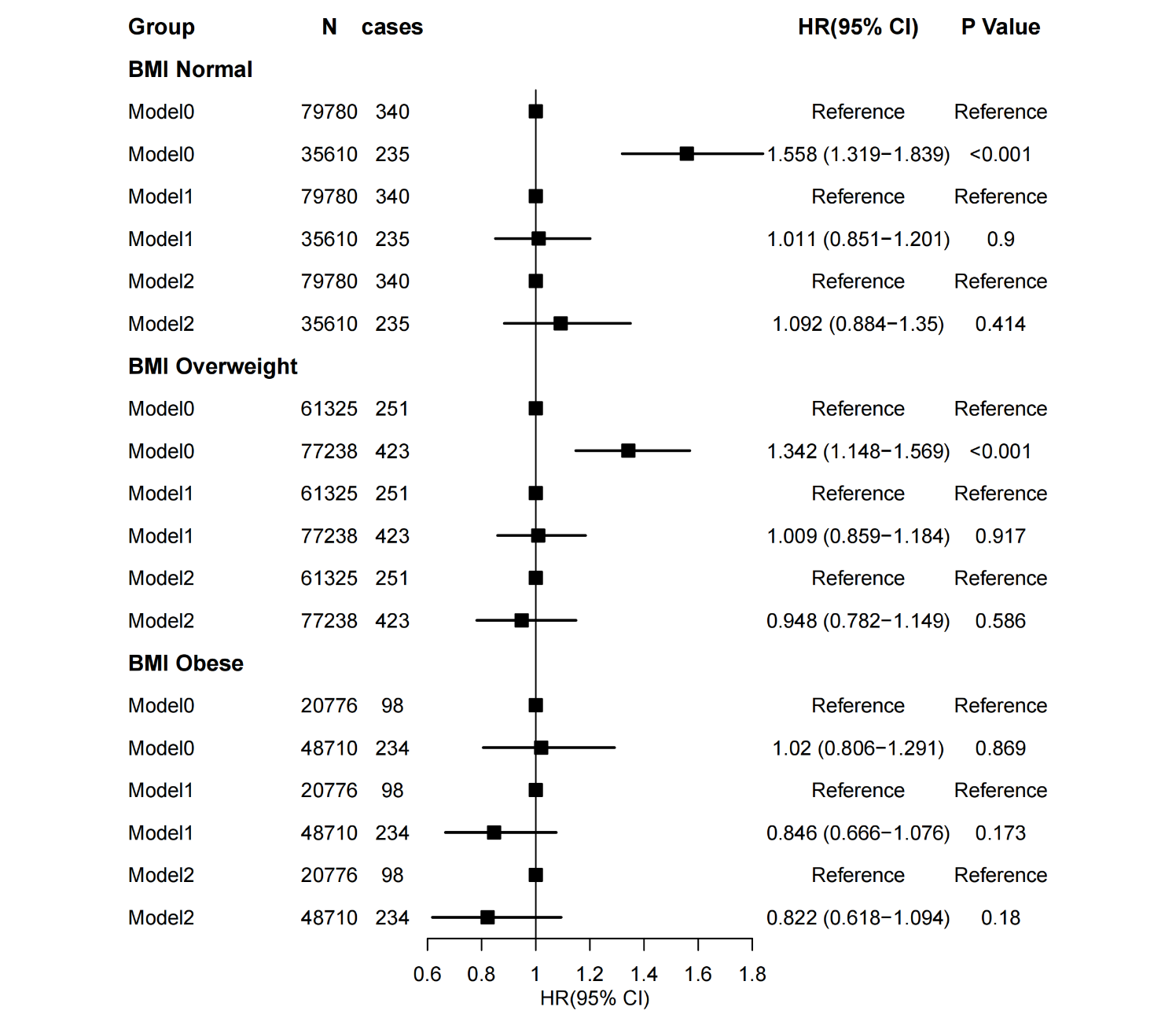

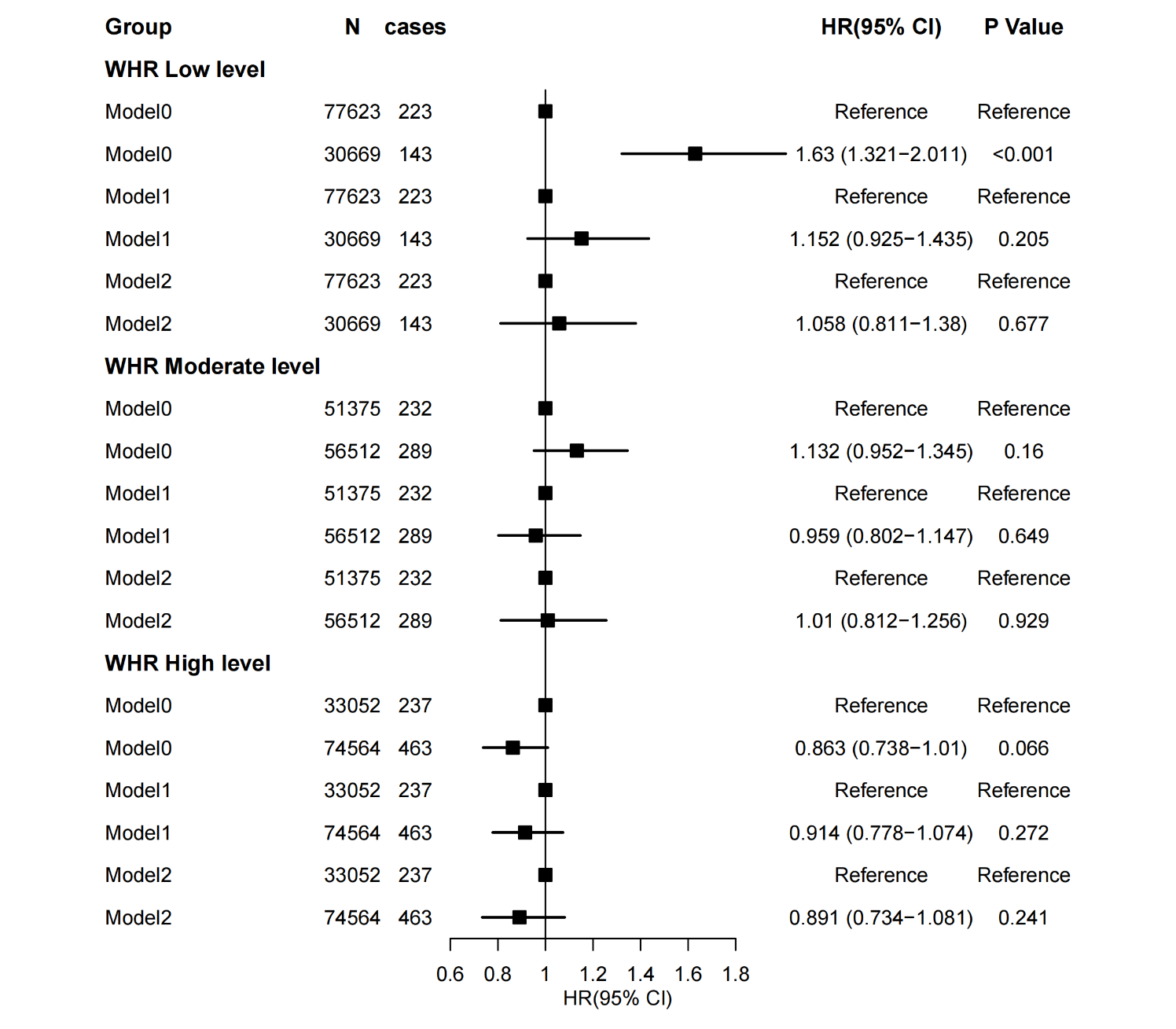

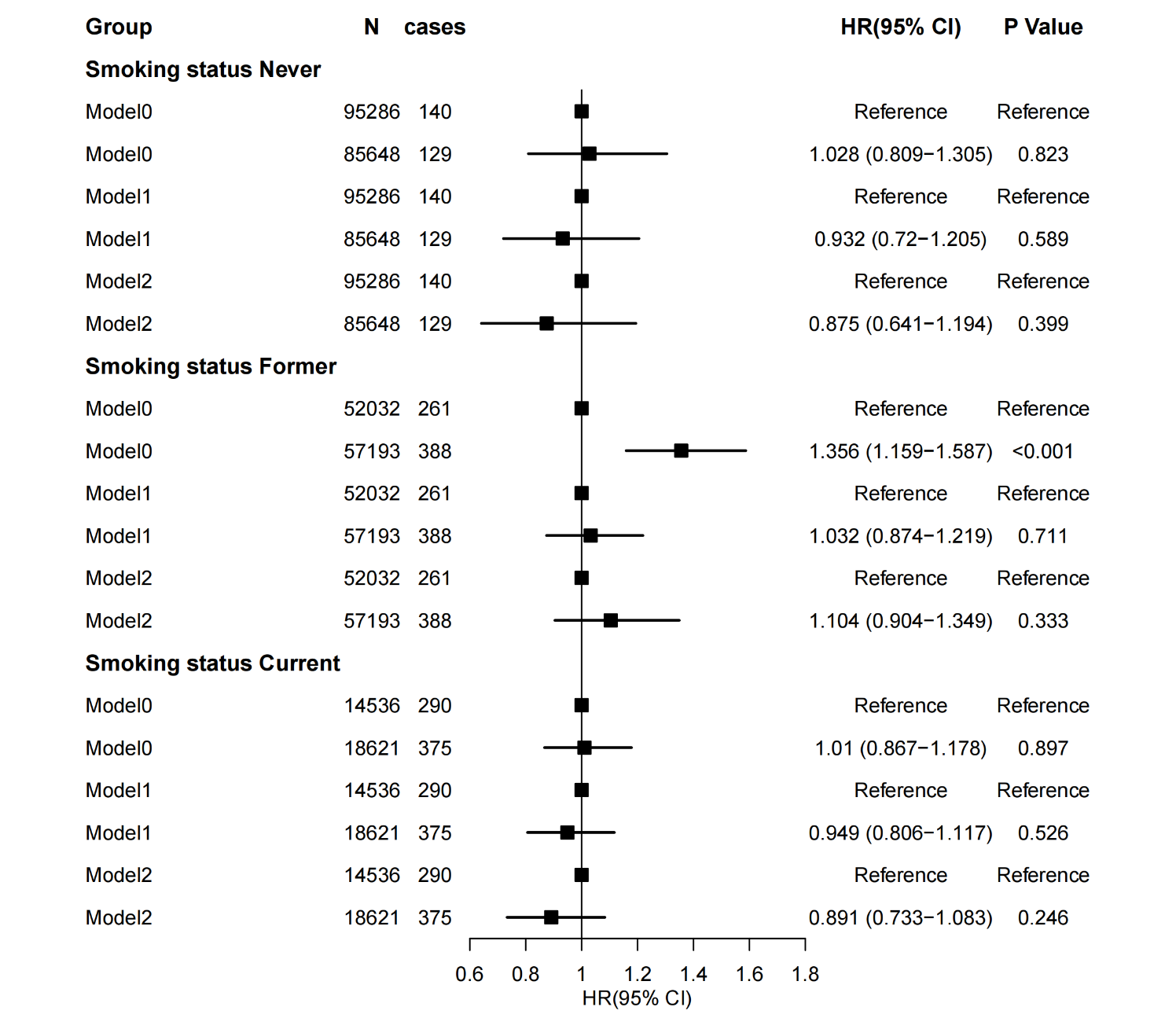


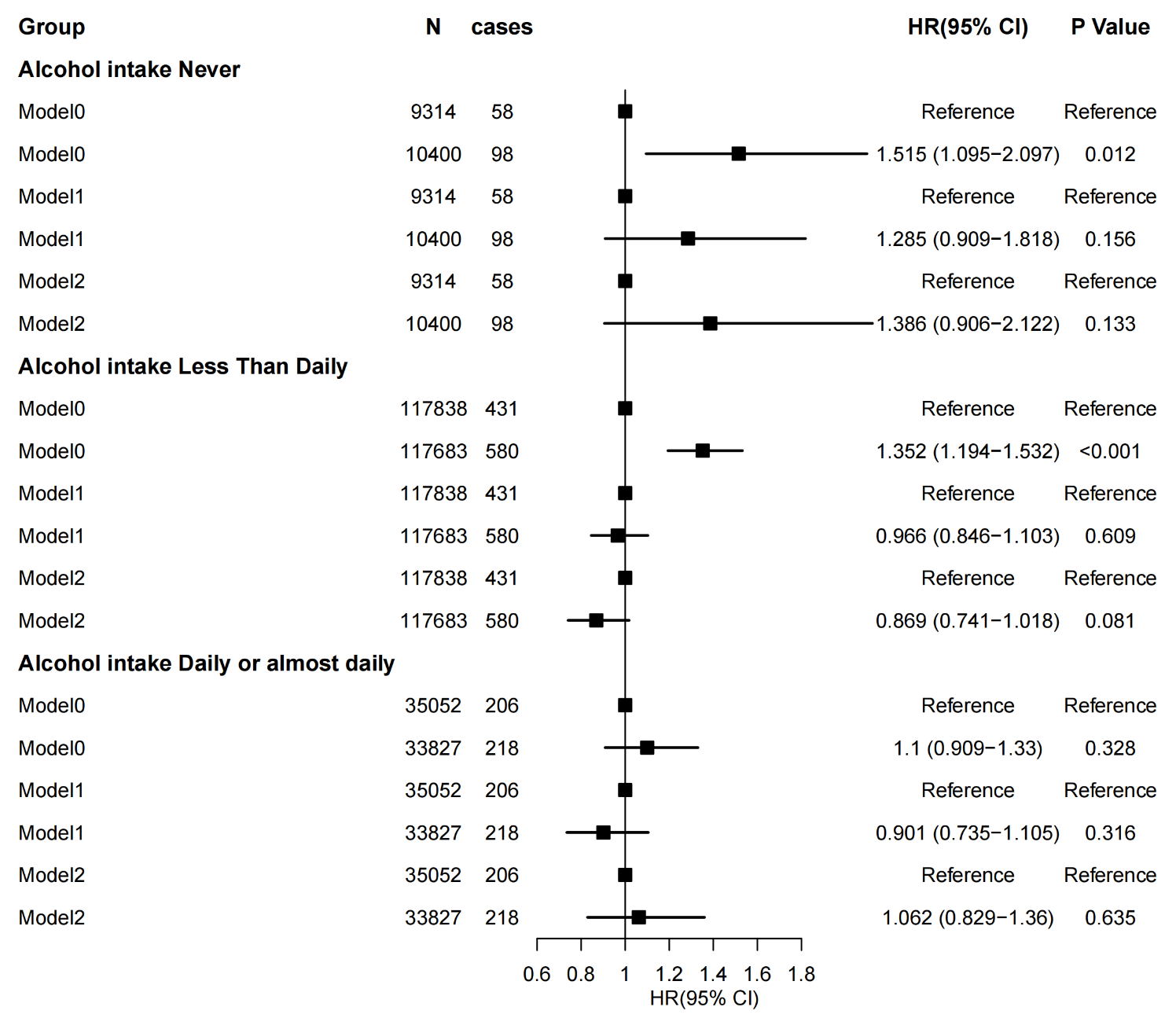


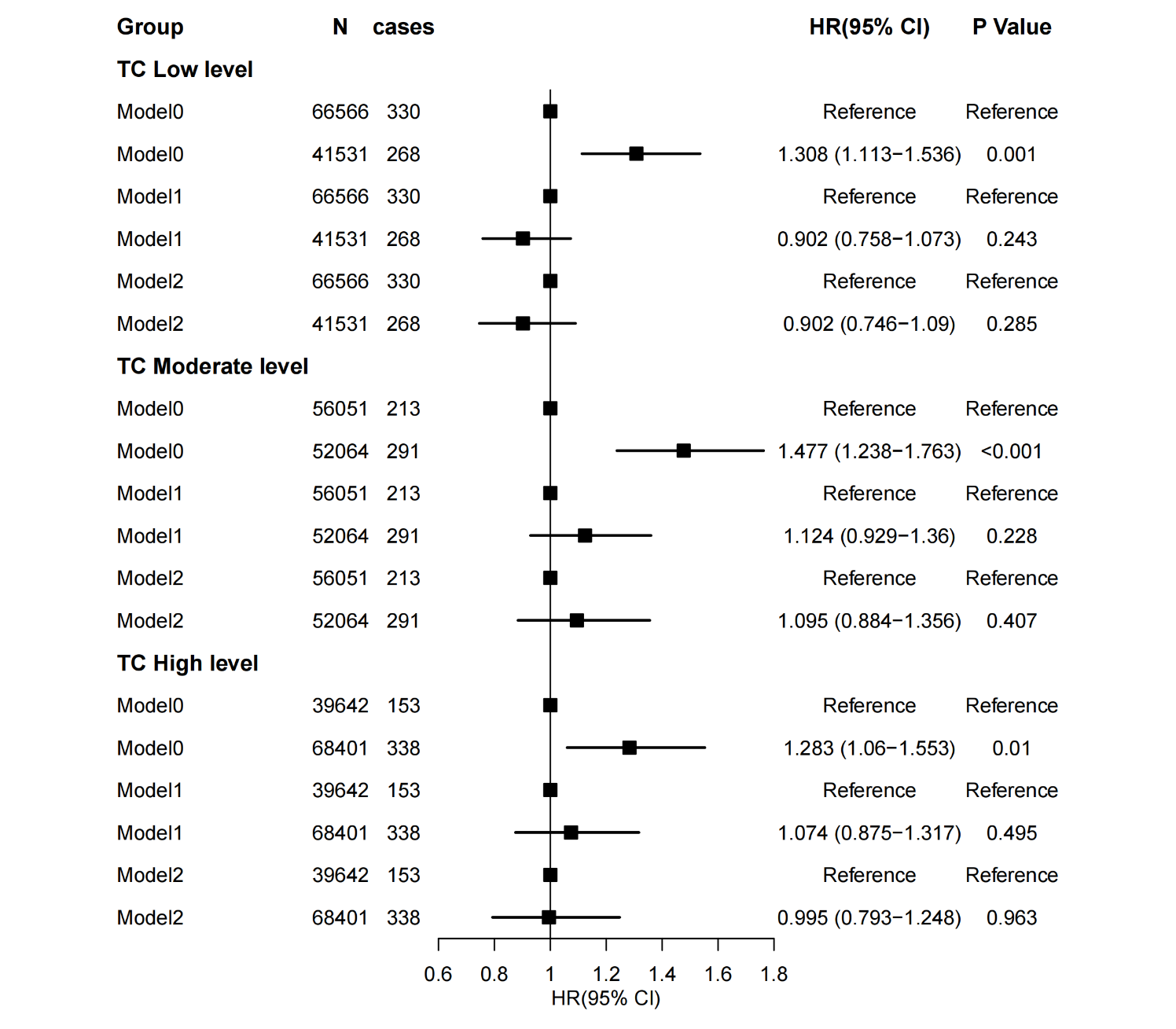

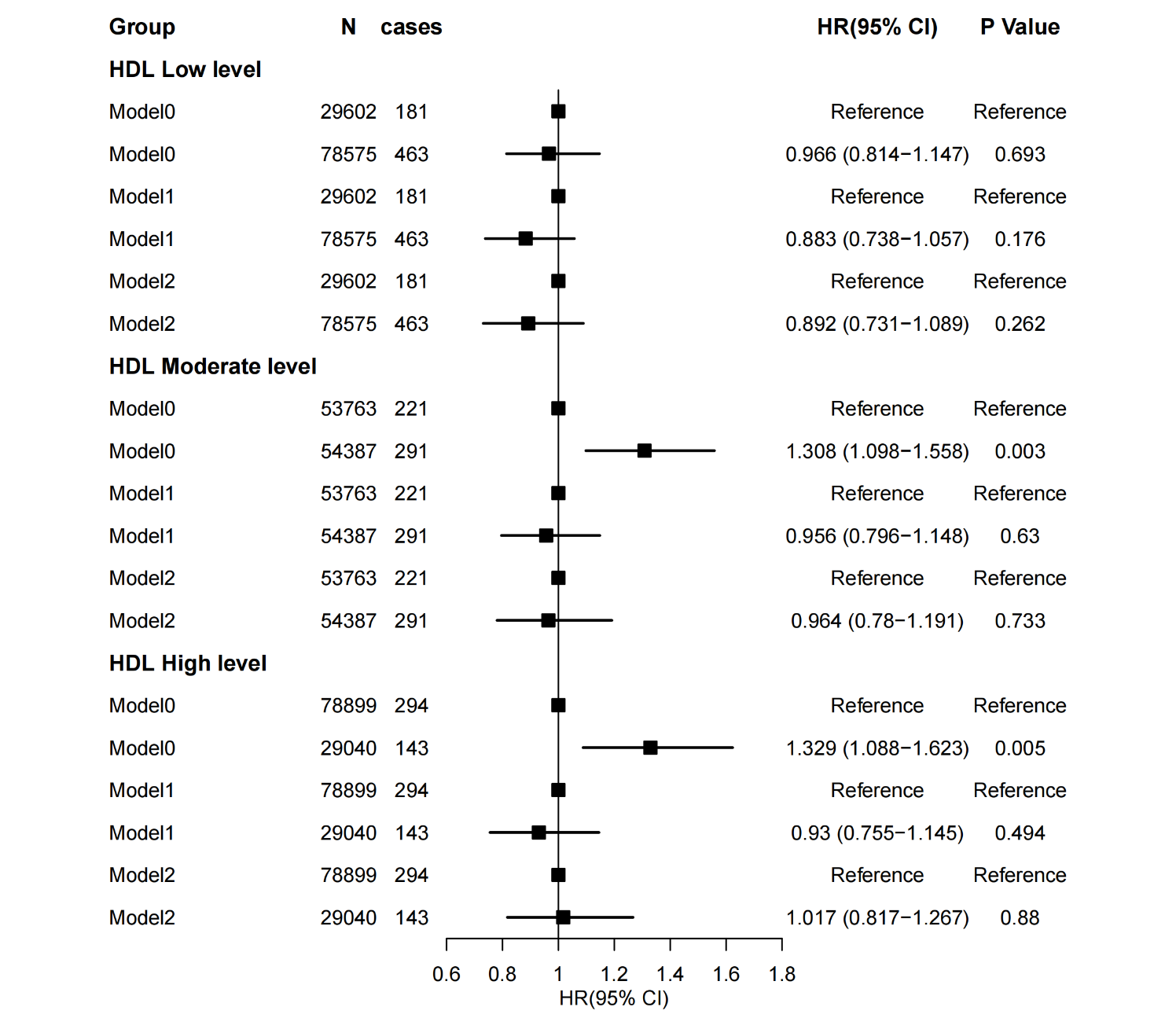

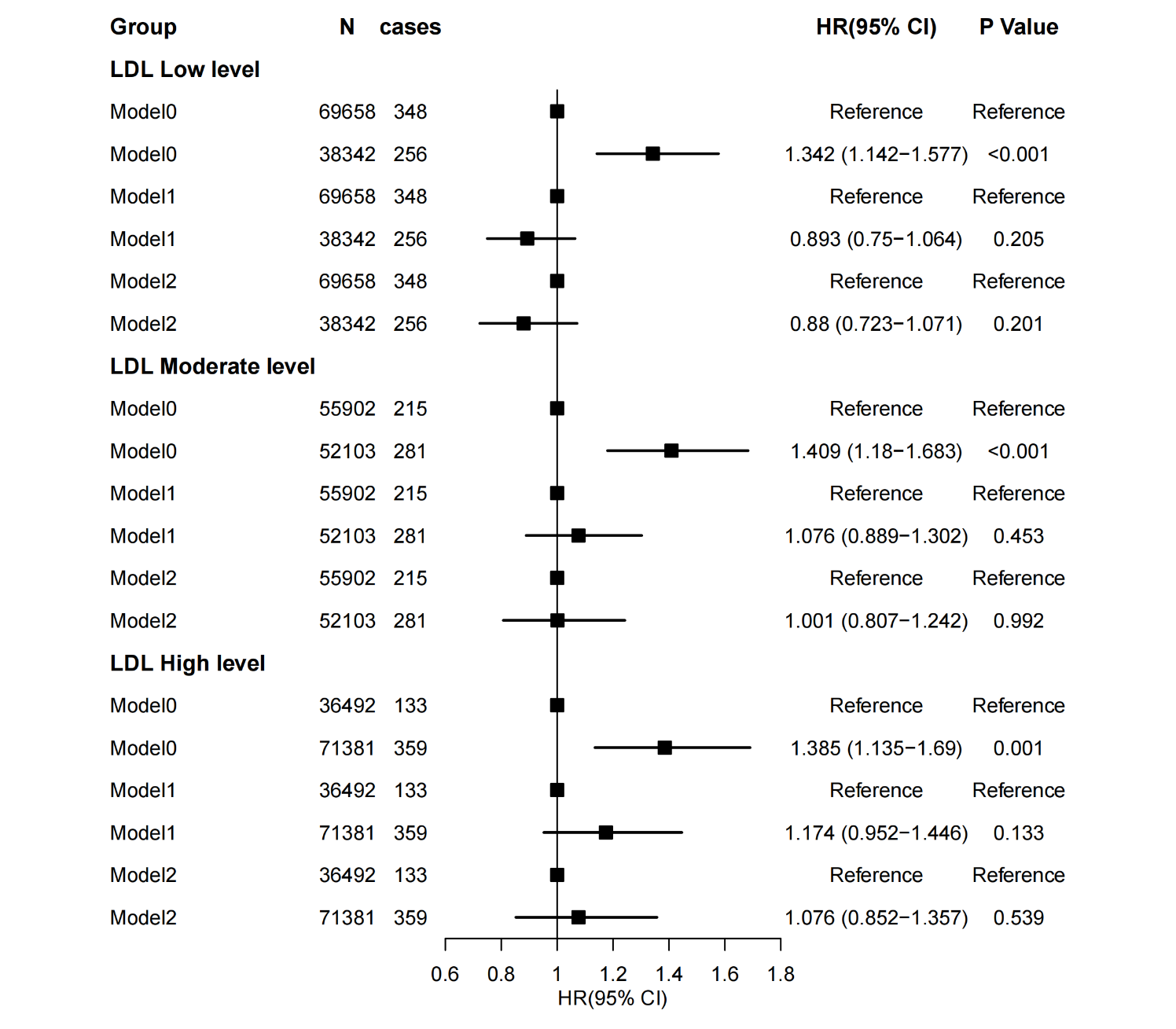

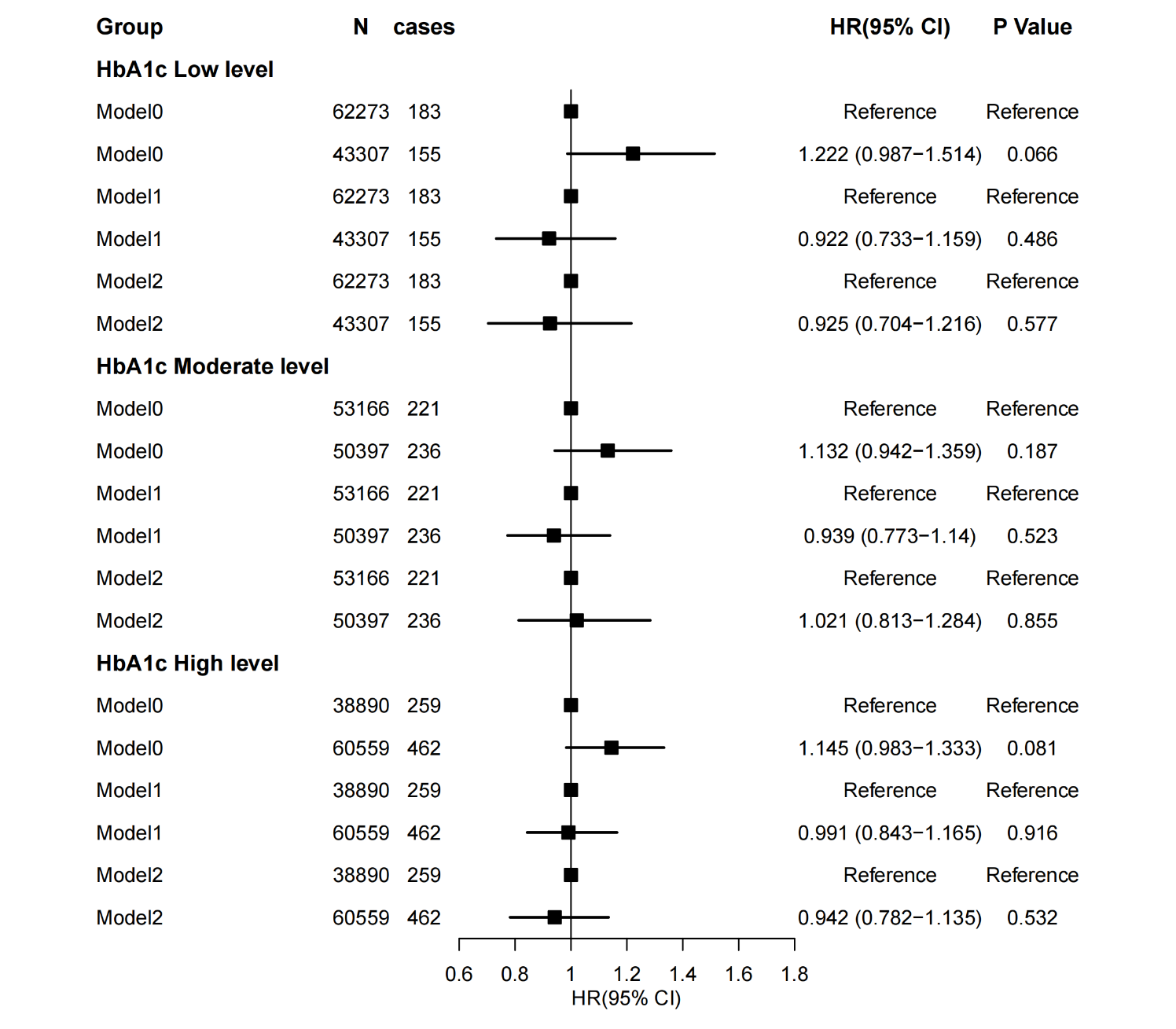

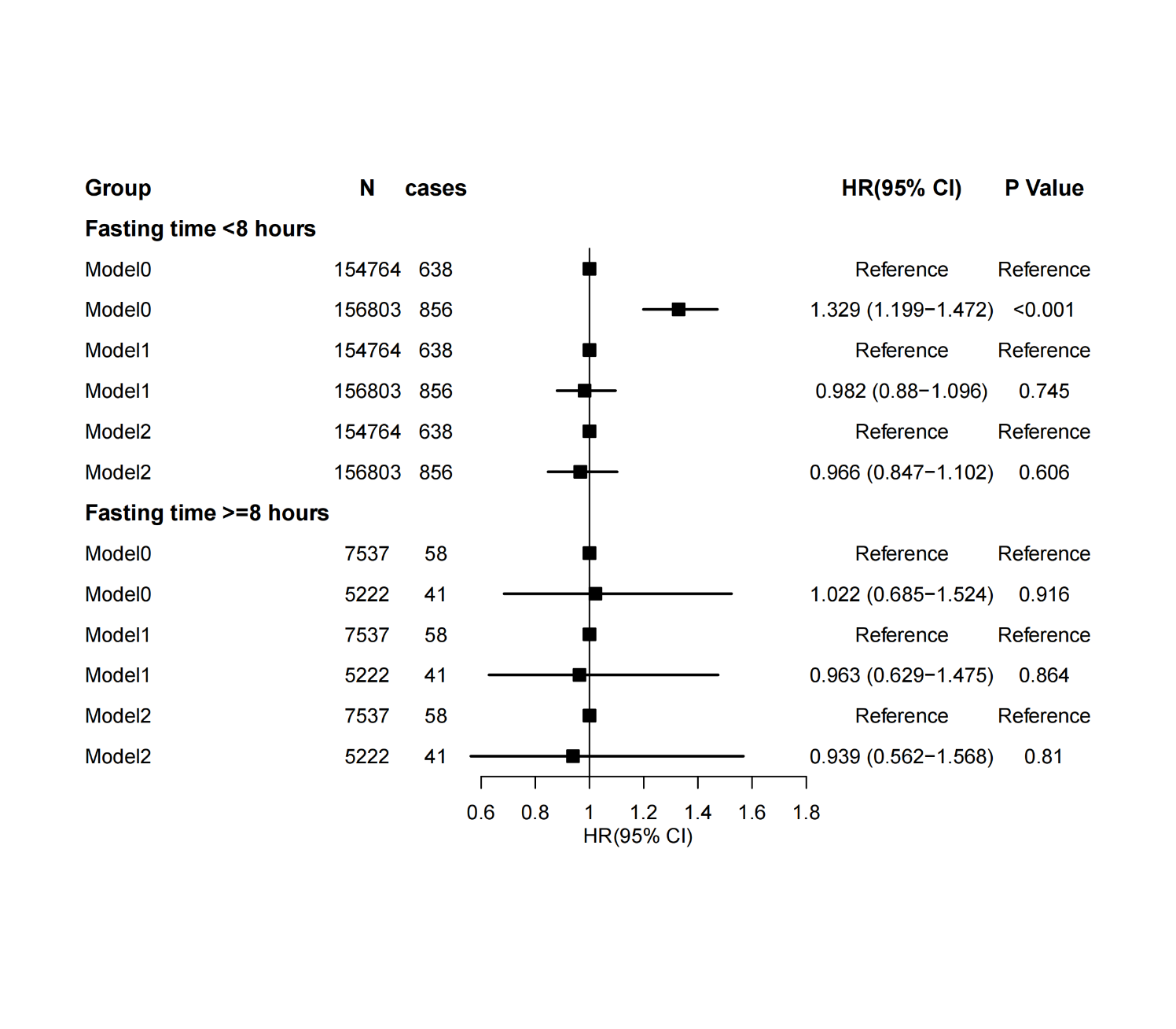

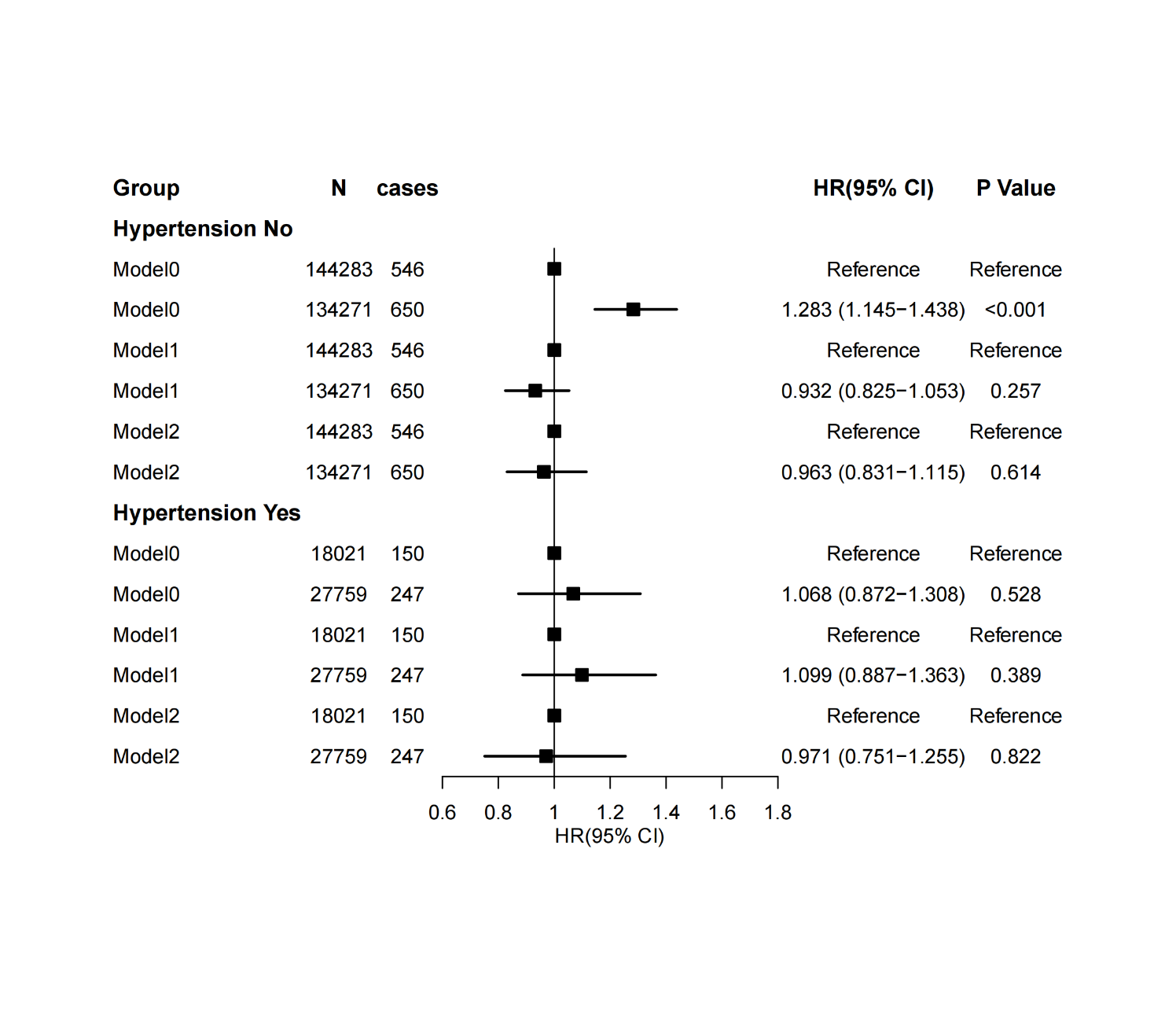


**Supplementary Figure 2.**

Forest plot of Cox regression model estimates for the association between the triglyceride-glucose index, which was divided by median level, and lung cancer risk under different subgroup analyses. Model 0: univariate Cox model; Model 1: adjusted for age, sex, region, Townsend deprivation score, smoking status, alcohol intake frequency, body mass index, waist hip ratio, and hypertension; Model 2: adjusted for Model 1 plus fasting time, total cholesterol, low-density lipoprotein cholesterol, high-density lipoprotein cholesterol, and glycated hemoglobin.


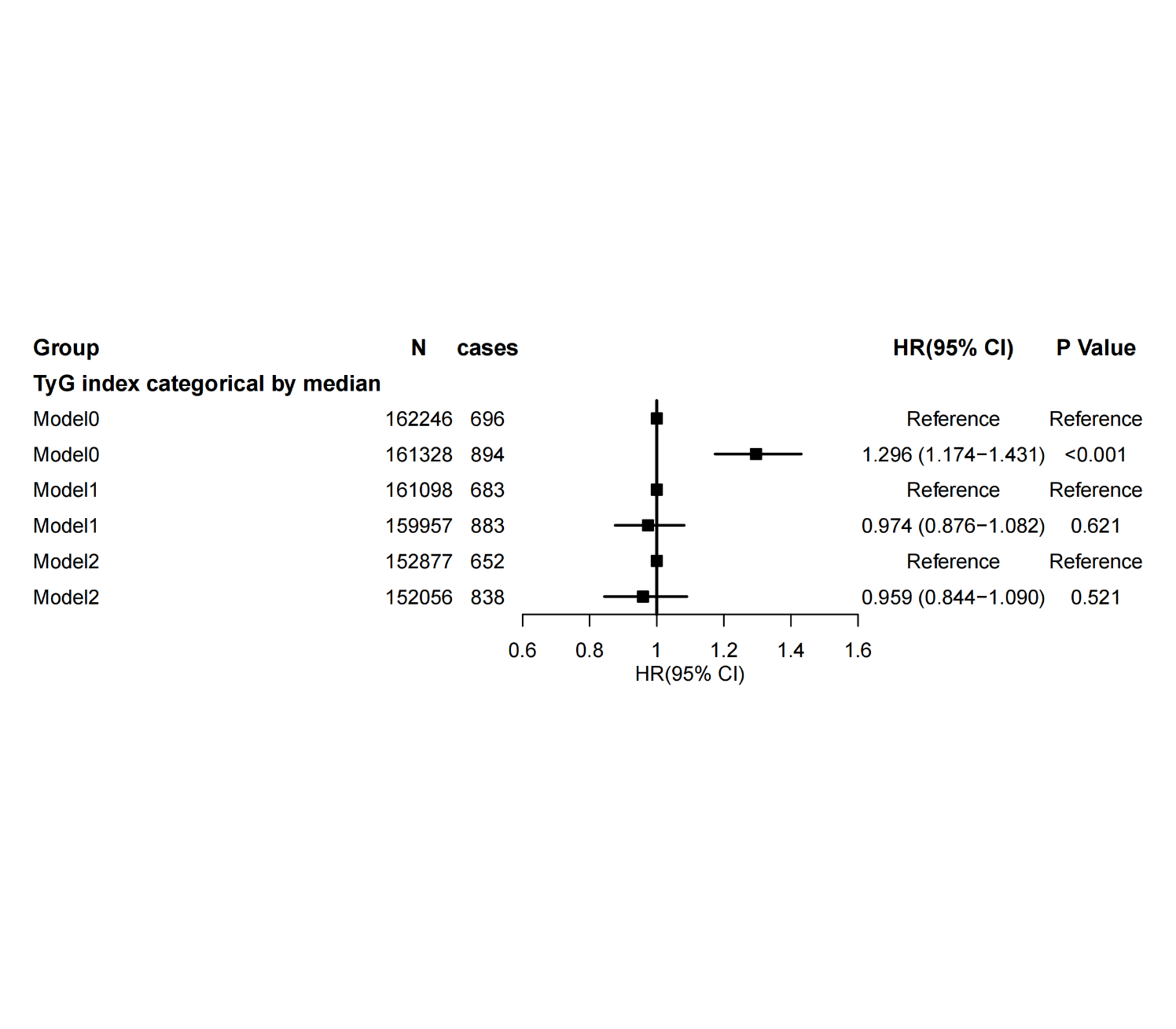


**Supplementary Figure 3**.

Forest plot of Cox regression model estimates for the association between the triglyceride-glucose index, which was divided by median level, and lung cancer risk after excluding individuals whose triglyceride-glucose index was beyond the range of mean ± 3SD in sensitivity analyses. Model 0: univariate Cox model; Model 1: adjusted for age, sex, region, Townsend deprivation score, smoking status, alcohol intake frequency, body mass index, waist hip ratio, and hypertension; Model 2: adjusted for Model 1 plus fasting time, total cholesterol, low-density lipoprotein cholesterol, high-density lipoprotein cholesterol, and glycated hemoglobin.


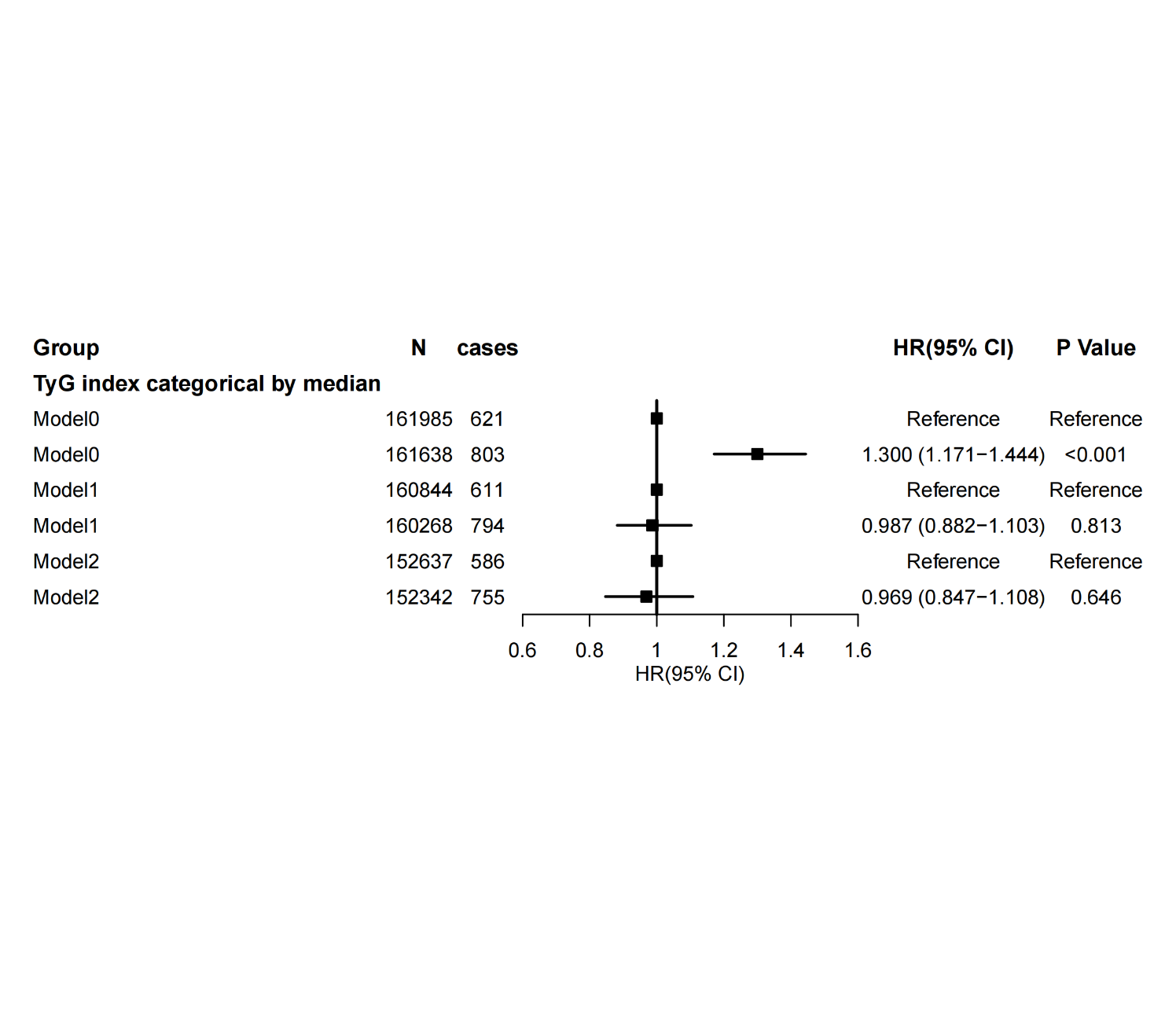


**Supplementary Figure 4**.

Forest plot of Cox regression model estimates for the association between the triglyceride-glucose index, which was divided by median level, and lung cancer risk after excluding individuals whose follow-up time less than 2 years in sensitivity analyses. Model 0: univariate Cox model; Model 1: adjusted for age, sex, region, Townsend deprivation score, smoking status, alcohol intake frequency, body mass index, waist hip ratio, and hypertension; Model 2: adjusted for Model 1 plus fasting time, total cholesterol, low-density lipoprotein cholesterol, high-density lipoprotein cholesterol, and glycated hemoglobin.

**
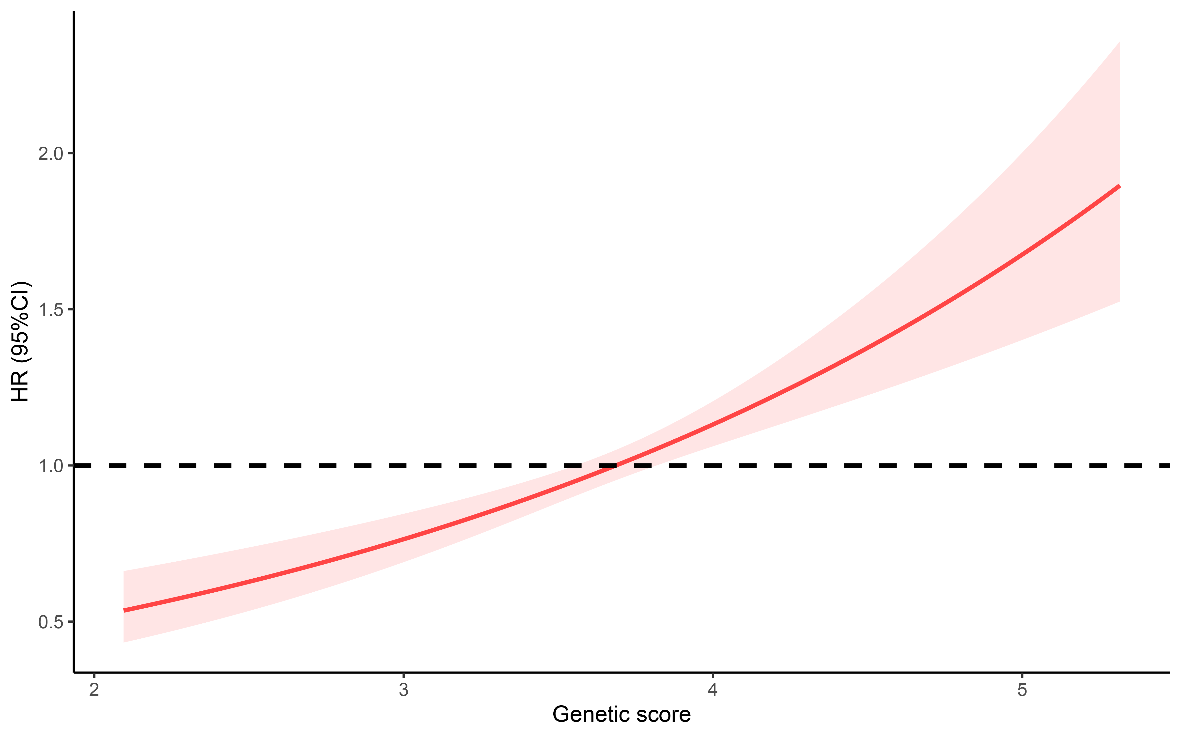
**

**Supplementary Figure 5.**

The relationships between polygenic risk score and lung cancer risk in the UK Biobank.


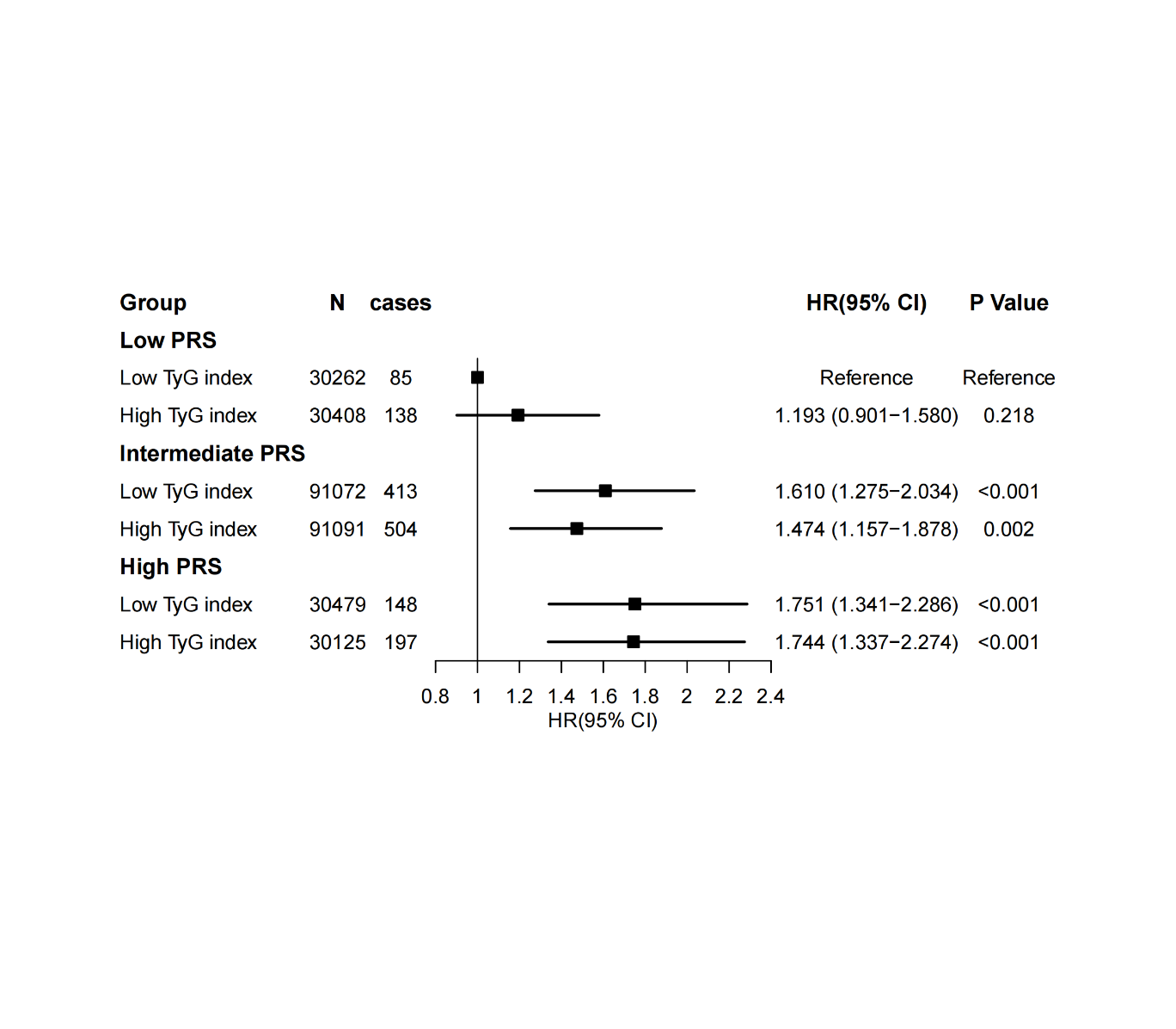


**Supplementary Figure 6.**

Risk of incident lung cancer according to genetic risk and TyG index categories. Participants were divided into categories of low (quintile 1), intermediate (quintiles 2–4), and high (quintile 5) genetic risk strata. TyG index was defined as low and high according to the median level (TyG index = 8.639). The hazard ratios were estimated using Cox regression models after adjusting for age, sex, region, Townsend deprivation score, smoking status, alcohol intake frequency, body mass index, waist hip ratio, and hypertension, fasting time, total cholesterol, low-density lipoprotein cholesterol, high-density lipoprotein cholesterol, and glycated hemoglobin, as well as the top 10 principal components of the ancestry and genotyping array.

TyG, triglyceride-glucose index

## Supplementary Tables

Table S1. Data collection and categorization of variables

| **Class** | **Variable** | **Description** | **Groups** |
| --- | --- | --- | --- |
| Demographic | Age | Continuous (unit, year) | Continuous |
| Demographic | Sex | Categorical | Female;Male |
| Demographic | Region | London: Barts,Hounslow,Croydon; Wales: Wrexham,Swansea,Cardiff; North West: Stockport pilot,Manchester,Liverpool,Bury; North East: Newcastle,Middlesborough; Yorkshier and Humber: Leeds,Sheffield; West Midlands: Stoke,Birmingham; East Midlands: Nottingham; South East: Oxford,Reading; South West: Bristol; Scotland: Glasgow,Edinburgh | London;Wales;North West;North East;Yorkshier and Humber;West Midlands;East Midlands;South East;South West;Scotland |
| Demographic | Townsend deprivation index | Categorical, quartile | Q1;Q2;Q3;Q4 |
| Body | BMI | <25, 25-30,>=30 (unit, kg/m^2^) | Normal;Overweight;Obese |
| Body | WHR | Categorical, quartile | Q1;Q2;Q3;Q4 |
| Smoking | Smoking Status | Never;Previous;Current | Never;Previous;Current |
| Alcohol intake frequency | Alcohol intake frequency | never, special occasions only, 1-3 times/month, 1-2 times/week, 3 or 4 times/week, and daily or almost daily | never, special occasions only, 1-3 times/month, 1-2 times/week, 3 or 4 times/week, and daily or almost daily |
| Blood index | Fasting time | Continuous (unit, hour) | Continuous |
| Blood index | TC | Cholesterol (unit, mmol/L) | Continuous |
| Blood index | TG | Triglycerides (unit, mmol/L) | Continuous |
| Blood index | GLU | Glucose (unit, mmol/L) | Continuous |
| Blood index | HDL-C | High Density Lipoprotein cholesterol (unit, mmol/L) | Continuous |
| Blood index | LDL-C | Low density lipoprotein cholesterol direct (unit, mmol/L) | Continuous |
| Blood index | HbA1c | Glycated haemoglobin (unit, mmol/mol); Categorical, <42, ≥42 | Normal, high |
| [Disease]--Previous cancer | Previous cancer | ICD10 code: C00-C97, except C34 | No;Yes |
| [Disease]--Lung cancer | Lung cancer | ICD10 code: C34 | No;Yes |
| [Disease]--Endocrine | Diabetes | ICD10 code: E10-E14 | No;Yes |
| [Disease]--Endocrine | Dyslipidemia | ICD10 code: E78 | No;Yes |
| [Disease]--Cardiac Disease | Hypertension | ICD10 code: I10-I15 | No;Yes |

Table S2. SNPs list of lung cancer identified in the study of McKay et al.

| **chr** | **SNP** | **Reference** | **Effect** | **EAF** | **OR** | **95%CI** |
| --- | --- | --- | --- | --- | --- | --- |
| 1 | rs71658797 | T | A | 0.103 | 1.13 | 1.09-1.18 |
| 3 | rs13080835 | G | T | 0.493 | 0.9 | 0.87-0.92 |
| 5 | rs7705526 | C | A | 0.342 | 1.25 | 1.21-1.29 |
| 6 | rs3094604 | A | G | 0.155 | 1.25 | 1.19-1.32 |
| 6 | rs6920364 | G | C | 0.456 | 1.07 | 1.05-1.10 |
| 8 | rs11780471 | G | A | 0.06 | 0.87 | 0.83-0.91 |
| 8 | rs4236709 | A | G | 0.218 | 1.13 | 1.09-1.18 |
| 9 | rs885518 | A | G | 0.101 | 1.17 | 1.11-1.23 |
| 10 | rs11591710 | A | C | 0.137 | 1.16 | 1.11-1.22 |
| 11 | rs1056562 | C | T | 0.473 | 1.11 | 1.07-1.14 |
| 12 | rs7953330 | G | C | 0.315 | 0.86 | 0.83-0.90 |
| 13 | rs11571833 | A | T | 0.011 | 1.6 | 1.43-1.80 |
| 15 | rs55781567 | C | G | 0.367 | 1.3 | 1.27-1.33 |
| 15 | rs77468143 | T | G | 0.253 | 0.86 | 0.83-0.89 |
| 15 | rs66759488 | G | A | 0.362 | 1.07 | 1.05-1.10 |
| 19 | rs56113850 | C | T | 0.44 | 0.88 | 0.86-0.91 |
| 20 | rs41309931 | G | T | 0.117 | 1.17 | 1.11-1.23 |
| 22 | rs17879961 | A | G | 0.005 | 0.41 | 0.32-0.52 |

Abbreviation: SNP, single nucleotide polymorphisms; chr, chromosome; EAF, effect allele frequency; OR, odds ratio; CI, confidence interval
